# Extracellular vesicle IL-32 promotes the M2 macrophage polarization and metastasis of esophageal squamous cell carcinoma via FAK/STAT3 pathway

**DOI:** 10.1186/s13046-022-02348-8

**Published:** 2022-04-15

**Authors:** Yixuan Sun, Yuzhen Qian, Chunxia Chen, Hongfei Wang, Xiuman Zhou, Wenjie Zhai, Lu Qiu, Xiaowen Zhou, Haoming Ning, Yumiao Zhao, Chao Shi, Lu Han, Yuanming Qi, Yahong Wu, Yanfeng Gao

**Affiliations:** 1 School of Life Sciences, Zhengzhou University, Zhengzhou, 450001 China; 2 School of Pharmaceutical Sciences (Shenzhen), Sun Yat-sen University, Shenzhen, 518107 China; 3 International Joint Laboratory for Protein and Peptide Drugs of Henan Province, Zhengzhou University, Zhengzhou, 450001 China; 4 Henan Key Laboratory of Bioactive Macromolecules, Zhengzhou University, Zhengzhou, 450001 China; 5 Affiliated Cancer Hospital of Zhengzhou University and Henan Cancer Hospital, Zhengzhou, 450008 China

**Keywords:** Esophageal squamous cell carcinoma, IL-32, Extracellular vesicle, M2 macrophage polarization, Metastasis

## Abstract

**Background:**

Metastasis is the leading cause of mortality in human cancers, including esophageal squamous cell carcinoma (ESCC). As a pro-inflammatory cytokine, IL-32 was reported to be a poor prognostic factor in many cancers. However, the role of IL-32 in ESCC metastasis remains unknown.

**Methods:**

ESCC cells with ectopic expression or knockdown of IL-32 were established and their effects on cell motility were detected. Ultracentrifugation, Transmission electron microscopy and Western blot were used to verify the existence of extracellular vesicle IL-32 (EV-IL-32). Coculture assay, immunofluorescence, flow cytometry, and in vivo lung metastasis model were performed to identify how EV-IL-32 regulated the crosstalk between ESCC cells and macrophages.

**Results:**

Here, we found that IL-32 was overexpressed and positively correlated to lymph node metastasis of ESCC. IL-32 was significantly higher in the tumor nest compared with the non-cancerous tissue. We found that IL-32β was the main isoform and loaded in EV derived from ESCC cells. The shuttling of EV-IL-32 derived from ESCC cells into macrophages could promote the polarization of M2 macrophages via FAK-STAT3 pathway. IL-32 overexpression facilitated lung metastasis and was positively correlated with the proportion of M2 macrophages in tumor microenvironment.

**Conclusions:**

Taken together, our results indicated that EV-IL-32 derived from ESCC cell line could be internalized by macrophages and lead to M2 macrophage polarization via FAK-STAT3 pathway, thus promoting the metastasis of ESCC. These findings indicated that IL-32 could serve as a potential therapeutic target in patients with ESCC.

**Supplementary information:**

The online version contains supplementary material available at 10.1186/s13046-022-02348-8.

## Background

Esophageal cancer is the sixth cause of death in human cancer, among which approximately 90% is esophageal squamous cell carcinoma (ESCC) [1]. So far, the traditional therapeutic strategies can hardly improve the overall survival of ESCC, such as surgery, radiotherapy and chemotherapy [2]. Therefore, it is of great clinical value to discover novel therapeutic targets and prognostic markers of ESCC.

It is reported that inflammation is a crucial factor to establish a local pathological microenvironment, and some cytokines could promote the progression of tumor and attenuate the host antitumor response [3–5]. IL-32 was reported as a pro-inflammatory cytokine which has several isoforms, including IL-32α, IL-32β, IL-32γ and IL-32δ [6, 7]. IL-32 can interact with multiple proteins, including proteinase-3, integrin, paxillin and focal adhesion kinase (FAK) [8–10]. However, the receptor for IL-32 has not been clearly identified yet. It has been reported that IL-32 could promote the secretion of TNFα, IL-1β, IL-6 and IL-8 by activating NF-κB and p38-MAPK pathways. Han et al. had focused on the expression of IL-32 in CD45^+^ immune cells and found Treg cells overexpressed IL-32 in ESCC, but the function of IL-32 in tumor cells and macrophages was not mentioned [11]. Ma et al. found that high expression of IL-32 synergized with irradiation promoted the apoptosis of ESCC cells by inhibiting the STAT3 pathway in vitro [12]. But it is also unknown whether IL-32 communicates with immune cells. Though the over-expression of IL-32 was found in gastric cancer, lung cancer, breast cancer and esophageal cancer [13–17], the specific roles of IL-32 in ESCC cells remains largely unknown.

Macrophages show unique plasticity and heterogeneity, and they are not only essential for host immunity but also involved in the progression of cancer [18]. Macrophages can be polarized to the immune suppressive M2 macrophages by some cytokines, such as IL-8, which promoted the metastasis of cancers [19, 20]. Previous studies showed that recombinant IL-32 promoted M2 polarization in Mycosis fungoides and macrophage infected with HIV in vitro [21, 22]. However, the relationship between tumor cell-derived IL-32 and macrophage in ESCC remain unclear and require further research, and the pathway involved in this process also remain unclear.

EV are one of the important pathways for cell-cell communication in the tumor microenvironment [23]. The compartmental exchange among cells through EV has emerged as a central mechanism, which illustrated the complex communication between malignant and immunocytes during tumor initiation and progression [24, 25]. Evidences showed that EV play an important role in tumor metastasis via transferring proteins, miRNAs and lncRNAs, which could be internalized into recipient cells and modulate the function of these cells. In addition, previous studies have provided evidences that miRNAs loaded into EV could skew the phenotype of macrophages when it was taken up by macrophages [26, 27]. However, the mechanism of M2 macrophage polarization by EV in ESCC has not been elucidated yet, especially EV-derived cytokines.

In the present study, we analyzed the expression and pathological pattern of IL-32 via RNA microarray (84 paired ESCC tumor and peritumor tissues) and tissue microarray (56 paired ESCC tumor and peritumor tissues). Through establishing IL-32 overexpression and knockdown ESCC cell lines, we firstly identified that IL-32 was loaded in the EV through a non-classical secretory pathway in ESCC. Then, we found the expression of IL-32 was positively correlated with M2 macrophage. Through immunofluorescence and coculture assays, we studied whether IL-32 in ESCC-derived EV could affect the phenotype and function of macrophages, as well as the underlying mechanisms. This could help us to understand the role of EV-IL-32 derived from ESCC in promoting the M2 macrophage polarization and the metastasis of ESCC.

## Methods

### Tissue samples and clinicopathologic parameters

ESCC samples (84 paired tumor and peritumor tissues) were obtained from the Affiliated Cancer Hospital of Zhengzhou University. Paired tissue samples were from ESCC patients of the stage IA-IB (*n* = 26), IIA-IIB (*n* = 29), IIIA-IIIC (*n* = 29). None of the patients had previously received chemotherapy, radiotherapy or other therapies, and all patients underwent an informed consent process. Sample collection and the biological experiments in this study were approved by the Ethics Committee of Zhengzhou University (ZZUIRB2021-32). The clinicopathologic parameters were analyzed according to age, gender, stage, tumor differentiation, and lymph node metastasis.

### Tissue microarray and immunohistochemistry (IHC) staining assay

Primary tumor and the paired peritumor samples were collected for tissue microarrays. Tissue cores with a diameter of 1.0 mm were taken from the tumor samples by tissue array instrument (Beecher Instruments, Silver Spring, USA). Standard IHC procedures were performed as previously described [28]. The expression of IL-32 and CD206 in the tumor and paired peritumor tissues of the Paraffin-embedded samples were evaluated with anti-human IL-32 (1: 200, BioLegend, 513501, USA) and anti-human CD206 (1: 200, Servicebio, GB11062, China). This assay contained 56 paired samples and all samples of patients without receiving neoadjuvant therapy previously and during the surgery, and all patients underwent an informed consent process. The IHC-score was calculated by multiplying the percentage (P) of positive cells by the intensity (I), according to the formula: H-Score = ∑ (P × I) = (Percentage of cells of weak intensity × 1) + (Percentage of cells of moderate intensity × 2) + (Percentage of cells of strong intensity × 3).

### ESCC cell lines and cell culture

Human ESCC cell lines (KYSE150, KYSE450, EC1, EC109 and EC9706), and immortalized normal esophageal cell line Het-1 A were cultured in complete RPMI 1640 medium (Gibco, Grand Island, USA). Complete medium was supplemented with 10% fetal bovine serum (FBS, BI, USA), 100 U/mL penicillin (Solarbio, China) and 100 µg/mL streptomycin (Solarbio, China); and cells were cultured at 37 °C with 5% CO_2_ under fully humidified conditions. GW4869 (Sigma-Aldrich, D1692, USA) and Y15 (Topscience, T7119, China) were used in cell culture.

Monocyte-derived macrophages (MDMs) were generated from human peripheral blood mononuclear cells (PBMCs) as previously described [29]. Briefly, Human PBMCs were collected from venous blood of healthy volunteers, which were diluted with PBS (pH 7.4) and separated with Ficoll density gradient. The cells were resuspended at 2 × 10^6^ cells/mL in RPMI1640 with 10% FBS. The cells were seeded in culture dishes to adhere and incubated for 4 h at 37 °C. Non-adherent cells were washed with complete media. The adherent monocytes were cultured in the complete medium supplement with 50 ng/mL human M-CSF (Cat: AF-300-25, PeproTech) for 7 days. To obtain EV-IL-32-induced M2 macrophage, MDMs were treated with EV-IL-32 for 72 h.

### Identification the isoform of IL-32

Primers (Table S1) were used to amplify all isoforms of IL-32 in ESCC tumor tissues and cell lines. The PCR products were cloned into the T/A cloning vector pMD19-T. Vectors were amplified using Stbl3-competent cells. The monoclonal colonies were cultured and amplified. IL-32 isoforms were confirmed by DNA sequencing. To further certificate the results, different primers were designed and qRT-PCR was performed to distinguish each isoform. Primers were listed in the Table S1 and diagramed in the Fig. S3C.

### Plasmid construction and transfection

Lentivirus transfection system was used to establish the stable *IL-32β* overexpression KYSE150 (KYSE150 IL-32β) cells. The mRNA was extracted from tumor tissues of ESCC patients, and reverse-transcribed into the full-length *IL-32β* cDNA, which was cloned into the pLVX-puro vector through EcoR I and Xba I restriction sites. Subsequently, the *IL-32β* overexpression vector was transfected into KYSE150 cells with PowerTrans 293 (Sixiang Biological, SX-TR293-001, China) according to the protocol. Empty vector was transfected into KYSE150 cells as control (KYSE150 vector). Short hairpin RNA (shRNA) was cloned into pSicoR-GFP vector and then was transfected into EC109 (EC109 shIL-32) cell. Empty vector was transfected into EC109 cells as control (EC109 shNC). The target sequence is 5’-AGAGCTCACTCCTCTACTTGA-3’.

### Real-time quantitative reverse transcription PCR (qRT-PCR)

Total RNA was extracted from cell lines and tissue samples by the total RNA Isolation Kit (TIANGEN, DP419, China) according to the manufacturer’s instructions. The first-strand cDNA was synthesized from 2 µg of total RNA with the Revert Aid First Strand cDNA Synthesis Kit (Thermo Fisher Scientific, K1622, USA). qRT-PCR assay was performed by SYBR Green I Master (Roche, 04887352001, Switzerland) in Roche LightCycler 480 II. The primers for qRT-PCR were listed in the Table S2. *GAPDH* was used to normalize the data.

### MTT assay

The growth of EC109 shNC, EC109 shIL-32, KYSE150 vector and KYSE150 IL-32β cell lines were determined by MTT assay. Briefly, cells were seeded into 96-well culture plates at the density of 4000 cells/well. After 24, 48, and 72 h, cell viability was measured with MTT reagent (Sigma, M2003, USA). Formazan crystals were dissolved in 150 µL DMSO. The absorbance at 490 nm was measured by SpectraMax iD5 (Molecular Devices, USA).

### Migration and invasion assays

Cell migration was measured by transwell assays. EC109 shNC, EC109 shIL-32, KYSE150 vector and KYSE150 IL-32β cell lines were resuspended in serum-free RPMI 1640 (200 µL) at the density of 5 × 10^5^ cells/mL, which were seeded into upper chamber (Corning, 353097, USA). The lower chamber was added with complete RPMI 1640 medium. Cells was cultured at 37 °C with 5% CO_2_ for 48 h. The migration cells were fixed with 4% paraformaldehyde for 30 min. Finally, 0.2% crystal violet was used to stain the cells which migrated to the bottom of the well.

For cell invasion assay, the transwell assay with matrigel (BD, 354234, USA) was performed as previously described [30]. Briefly, 200 µL cell solution (EC109 shILNC, EC109 shIL-32, KYSE150 vector and KYSE150 IL-32β, 1 × 10^5^ cells/well) were seeded into the upper chamber (8 μm pore, Corning, 353097, USA) which was coated by the matrigel. The lower chambers were filed with 600 µL of RPMI 1640 medium including 10% FBS. After 48 h, invasion cells were fixed in 4% paraformaldehyde for 30 min, and stained with 0.2% crystal violet for 30 min.

### Wound healing assay

EC109 shNC, EC109 shIL-32, KYSE150 vector and KYSE150 IL-32β cell lines were seeded into 24-well plate in the serum-free RPMI 1640 with the number of 5 × 10^5^ cells/well cultured overnight. Cells were scratched by micropipette tip. After incubation for 12 or 24 h, the wound area was observed by microscope.

### Coculture assay

In the 24-well Transwell (8.0 μm aperture) co-culture system, EC109 and KYSE150 cell lines were seeded into the upper chamber with 1 × 10^5^/well and EV-IL-32-educated M2 macrophages into the lower chamber with the same density. After 48 h, migrated cells were fixed in 4% paraformaldehyde for 30 min, and stained with 0.2% crystal violet for 30 min.

### Enzyme-linked immunosorbent assay

Supernatant was collected from EC109 shNC, EC109 shIL-32, KYSE150 vector and KYSE150 IL-32β cell lines cultured for 48 h. Then, supernatant was centrifuged to eliminate the debris (600 g for 10 min) and large vesicles (10,000 g for 30 min). EV were treated by Native lysis Buffer (Solarbio, R0030, China). Human IL-32 DuoSet ELISA kit (R&D Systems, DY3040-05, USA) was used to measure the concentration of IL-32 according to the manufacturer’s instructions.

### Immunofluorescence (IF)

For CD206/IL-32/DAPI triple staining, slides were stained with rabbit anti-human CD206 (Servicebio, GB11062, China) and mouse anti-human IL-32 (BioLegend, 513501, USA) according to the manufacturer’s instructions. Sections were scanned by a TissueFAXS imaging system (Tissue Gnostics, Austria).

### Isolation and identification of EV

EV were isolated from cultured supernatant by differential centrifugation as previously described [31]. Briefly, supernatant was collected from cells that were cultured for 48 h in EV-free complete medium. Then, the supernatant was centrifuged at 300 g for 10 min, 3,000 g for 10 min to remove cells and debris, 10,000 g for 30 min to remove multivesicular. Subsequently, supernatant was ultra-centrifuged at 100,000 g for 2 h to obtain EV, and the EV were suspended in PBS (pH 7.2). Thereafter, the ultra-centrifugation step was repeated once. Finally, the supernatant was carefully removed and the EV were resuspended in 1 mL PBS (pH 7.2). The concentrations of EV proteins were measured by BCA assay.

Size distribution of the EV were identified by Malvern spray analyzer (Malvern Panalytical, England). The EV makers CD63 and TSG101 were detected by Western blot assay. For Transmission electron microscope (TEM) (HITACHI, Japan), EV were diluted in PBS (pH 7.2) and dropped onto a carbon-coated copper electron microscope grid for staining with uranium acetate.

### Labeling and tracking of EV

EV were stained with PKH67 (Umibio, UR52303. China) according to the manufacturer’s protocol. Briefly, 5 µL of PKH67 dye was mixed with 50 µL Diluent C to make the dye solution, which was added into EV for 10 min at room temperature. The stained EV were isolated according to the method mentioned above. To verify whether EV can be phagocytosed by macrophages, MDMs were cultured on 5 mm slides and stained with Hoechst 33342 (Beyotime, C1025, China) and Cellmask orange (Invitrogen, C10045, USA). The EV derived from EC109 were labeled with PKH67 and then cocultured with MDMs. The video was performed by LiT LBS Light-sheet Microscope.

### Flow cytometry

Subsets of M1 and M2 macrophages were defined as described previously [32]. Fluorophore-conjugated monoclonal antibodies specific to CD45, F4/80, CD11c, CD14, CD11b, CD80 and CD206 were listed in Table S3. Then, the phenotype and proportion were analyzed with the BD FACS Celesta flow cytometer (USA). Data were analyzed by FlowJo 7.6.

### Gene set enrichment analysis (GSEA)

The GSEA analysis was conducted with the cohort GSE23400 (Probe ID: 203828_s_at for IL-32). We divided the samples into two groups according to the median of IL-32 expression, and h.all.v6.0.synbols.gmt was obtained from the Molecular Signatures Database. RNA-Seq was also performed to analyze the pathway enrichment between IL-32 knockdown EC109 (EC109 shIL-32) and EC109 transfected with empty vector (EC109 shNC) by GSEA.

### Western blot

Total cell lysates were obtained with lysis buffer. Equal amounts of protein were separated by 10% SDS-PAGE gels and transferred to PVDF membrane (Merck Millipore, IPVH00010, USA). Then, PVDF membrane was blocked for 2 h by defatted milk (pH7.2 TBS containing 0.1% Tween 20 and 5% defatted milk) at 4 °C. Subsequently, the PVDF membranes were incubated with antibodies showed in Table S4. Blots were visualized by use of ECL system (Azure C600, USA).

### Mouse models

Subcutaneous tumor model was prepared as following. Six-week-old female BALB/c nude mice were subcutaneously injected on the right back with 3 × 10^6^ cells (KYSE150 vector, KYSE150 IL-32β, EC109 shNC and EC109 shIL-32). Tumor size and mouse body weight were measured every two days. After 18 days, all of the mice were sacrificed. Single-cell suspensions were made from tumor tissues and phenotypic analysis of macrophages was performed by flow cytometry.

For mouse lung metastasis models, six-week-old male nude BALB/c mice were intravenously injected with EC109 shNC or EC109 shIL32 (2 × 10^6^ cells/mouse). Seven weeks later, all of the mice were sacrificed. For KYSE150 mouse model, mice were inoculated *i.v.* with KYSE150 vector or KYSE150 IL-32β (1 × 10^6^ cells/mouse). Six weeks later, all of the mice were sacrificed. Lungs were removed and fixed by 4% formaldehyde. The number of the lung metastatic foci were counted, and tumor lesions within the lung tissues were confirmed by H&E staining.

For macrophages depletion, BALB/c nude mice were intravenously injected with 100 µL clodronate liposome or control liposome (FormuMax Scientific, USA) every 4 days after tail vein injection of 2 × 10^6^ EC109 shNC or EC109 shIL-32 cells until 7 weeks. The efficiency of macrophages depletion was determined by flow cytometry with analysis of the CD45^+^F4/80^+^CD11b^+^cells.

### Statistical analysis

Statistical analysis was performed using GraphPad Prism software version 8.0 (GraphPad Software Inc.). The correlation analysis between IL-32 expression and ESCC clinicopathological characteristics was determined by Chi-square test. Survival analysis was determined by the Kaplan–Meier method and differences between the groups were analyzed by means of log-rank test. Correlation analysis were performed by the Pearson’s rank correlation test. Statistical differences between two groups were evaluated with one-tailed unpaired or paired Student’s *t*-test. *P* < 0.05 is considered to be significant difference. The values were expressed as mean ± SEM.

## Results

### IL-32 is highly expressed in ESCC and positively correlated to poor prognosis

**Fig. 1:**
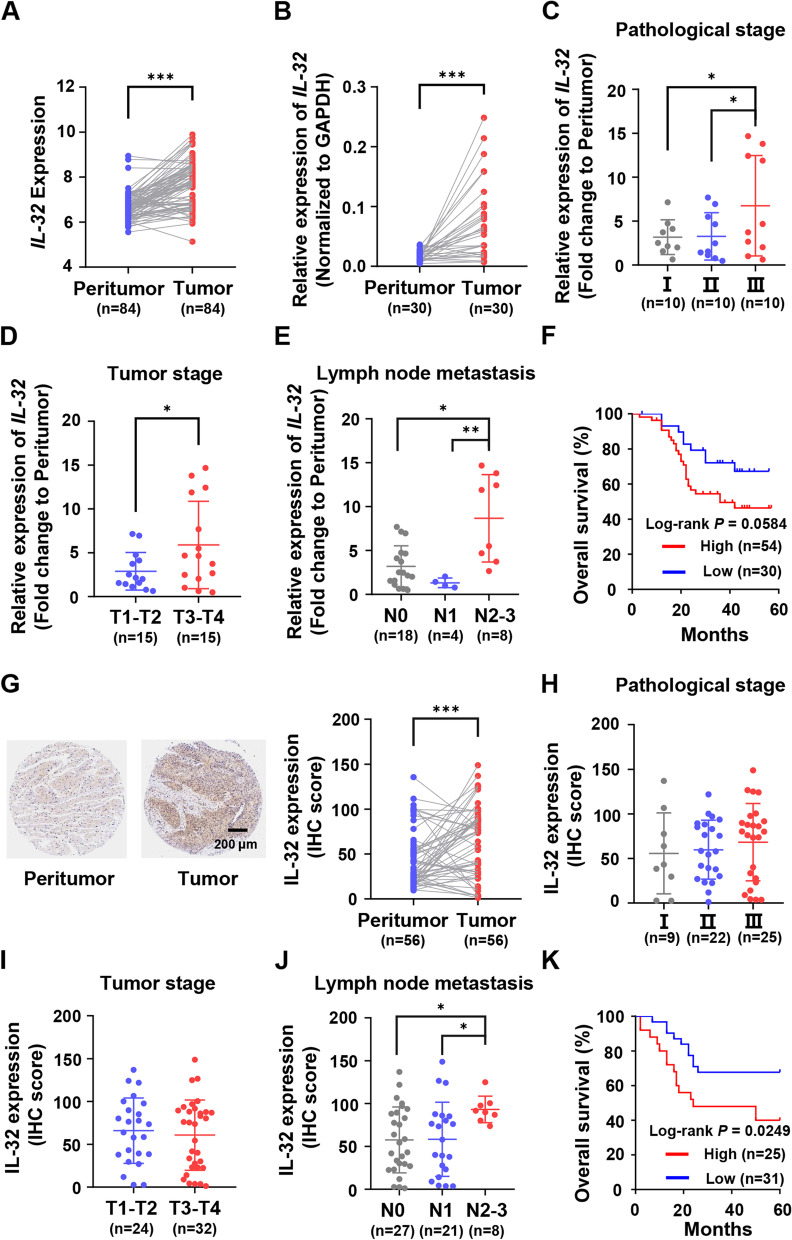
Expression and prognostic impact of IL-32 in human ESCC tissues with RNA and tissue microarray. **A** Normalized RNA microarray data of 84 paired ESCC tumor and peritumor tissues was used to analyze the mRNA expression of *IL-32* (Paired one-tailed Student’s *t*-test, ***P < 0.001). **B** *IL-32* mRNA levels were determined by qRT-PCR assay in 30 paired ESCC tumor and peritumor tissues from 84 paired ESCC patients (Paired one-tailed Student’s *t*-test, **P < 0.01). **C**-**E** Scatter plot of *IL-32*-fold change in tumor *versus* peritumor tissue was mapped according to pathological stage, tumor stage and lymph node metastasis (Student’s *t*-test, *P < 0.05, **P < 0.01). **F** Kaplan-Meier survival curves of high and low *IL-32* expression (Fold change to peritumor) groups of patients with ESCC defined by the cutoff value of 1.41 were established on the normalized RNA microarray data. (Log-rank *P* = 0.0584). **G** Representative IHC images of IL-32 in ESCC tumor and paired peritumor tissues (Scale bar, 200 μm). IHC scores of IL-32 in 56 paired ESCC tumor and peritumor tissues were analyzed and mapped (Paired one-tailed Student’s *t*-test, **P < 0.01). **H**-**J** Scatter plot of IL-32 IHC scores in tumor tissues were mapped according to pathological stage, tumor stage and lymph node metastasis (*n* = 56, Student’s *t*-test, *P < 0.05). **K** Kaplan-Meier survival curves of high and low IL-32 expression groups of patients with ESCC defined by the cutoff value of 80.01 established on the tissue microarray data (Log-rank *P* = 0.0249). Data are represented as means ± SEM

Firstly, we analyzed the expression difference of IL-32 between tumor tissues and the paired peritumor tissues by RNA microarray. We observed that the expression of *IL-32* was abnormally higher in ESCC tumor tissues (*n* = 84, *P* < 0.001) (Fig. 1A). We also analyzed the GEO cohorts (GSE23400, GSE20347 and GSE45670) and found that *IL-32* expression was higher in ESCC tumor tissues than peritumor tissues (Fig. S1A-C). To confirm this, 30 paired tumor and peritumor tissues from these 84 ESCC samples were randomly selected to detect the expression of *IL-32* by the way of qRT-PCR. The results also showed that *IL-32* was expressed higher in tumor tissues (*P* < 0.001) (Fig. 1B). To determine the association between *IL-32* expression and tumor characteristics, the fold change of *IL-32* in each group was analyzed with clinicopathologic parameters. Expression level of *IL-32* was positively correlated with the pathologic stage (*P* < 0.05), tumor stage (*P* < 0.05), as well as lymph node metastasis (*P* < 0.05, *P* < 0.01) (Fig. 1C-E). Among the 84 patients, the 5-year overall survival rate was acquired by dichotomization based on the cutoff value of *IL-32* expression level. The *IL-32* high expression group (*n* = 54) presented poorer survival (*P* = 0.058) compared with the *IL-32*-low expression group (*n* = 30) (Fig. 1F).

Subsequently, we analyzed the protein expression level of IL-32 by IHC tissue array, and other 56 paired tissue samples from ESCC patients were used. Consistent with the results of RNA microarray, strong staining of IL-32 was detected in the cytosol of tumor cells. In contrast, absent or weak staining of IL-32 was observed in the paired peritumor tissues (Fig. 1G). Then, the IHC score for IL-32 was analyzed with clinicopathologic parameters. Unlike the results of RNA microarray, the expression of IL-32 was not associated with the pathologic stage and tumor stage (Fig. 1H-I). Interestingly, higher IL-32 was detected in the advanced stages of lymph node metastasis (*P* < 0.05) (Fig. 1J). Survival analysis showed that the patients with higher IL-32 expression (*n* = 25) showed poorer survival compared with those patients with lower IL-32 expression (*n* = 31) (Fig. 1K). Some other clinicopathologic parameters, such as age, gender and tumor location, were analyzed in Table 1.

**Table 1:** Correlation of IL-32 expression level with clinicopathological features in ESCC patients

Variables	All cases (N)	IL-32 expression level (IHC-score)		*P* value^a, b^
		**Low, N (%)**	**High, N (%)**	
**Age (year)**				
< 57	15	9 (16.1)	6 (10.7)	0.905
>=57	41	22 (39.3)	19 (33.9)	
**Gender**				
Male	41	24 (42.9)	17 (30.4)	0.626
Female	15	7 (12.5)	8 (14.3)	
**Tumor location**				
Upper	9	8 (14.3)	1 (1.8)	0.087
Middle	33	16 (28.6)	17 (30.4)	
Lower	14	7 (12.5)	7 (12.5)	
**Tumor differentiation**				
Well	10	8 (14.3)	2 (3.6)	0.159
Moderate	33	15 (26.8)	18 (32.1)	
Poor	13	7 (12.5)	6 (10.7)	
**Pathological stage**				
IA-IB	9	6 (10.7)	3 (5.4)	0.304
IIA-IIB	22	14 (25)	8 (14.3)	
IIIA-IIIC	25	11 (19.6)	14 (25)	
**Tumor stage**				
T1-T2	24	13 (23.2)	11 (19.6)	0.265
T3-T4	32	18 (32.1)	14 (25)	
**Nodal stage**				
N0	27	18 (32.1)	9 (16.1)	0.025*
N1	21	12 (21.4)	9 (16.1)	
N2-N3	8	1 (1.8)	7 (12.5)	

*N* Number

^a^χ^2^ test to compare clinicopathological parameters in IL-32 low-expression group *versus* high-expression group

^b^*P* < 0.05 is compared significant

In summary, expression of IL-32 in ESCC tumor specimens was abnormally higher both in mRNA and protein levels, and the protein expression of IL-32 was positively correlated with lymph node metastasis, suggesting that overexpression of IL-32 may play an important role in the metastasis of ESCC.

### IL-32β is the main isoform in ESCC tissues and ESCC cell lines

To determine the main isoform of *IL-32* in ESCC tumor tissues and ESCC cell lines, we firstly used qRT-PCR to detect the expression of all *IL-32* isoforms in ESCC cell lines (KYSE150, KYSE450, EC1, EC109 and EC9706) and the normal esophagus epithelial Het-1 A cells (Fig. S2), and found that *IL-32* was highly expressed in EC109. Then, DNA sequencing was performed to accurately distinguish the isoforms of *IL-32* in ESCC specimens and EC109 cells. Our results showed that *IL-32β* was the main isoform expressed in ESCC specimens (65%) (Fig. S3A) and in EC109 (75%) (Fig. S3B). To further certificate this conclusion, different primers were designed and qRT-PCR was performed separately for each isoform (Table S1, Fig. S3C). The results showed that *IL-32β* was the main isoform and highly expressed in ESCC tumor tissues (*n* = 9) (Fig. S3D) and EC109 cell line (Fig. S3E). Moreover, the results of DNA sequencing showed that *IL-32θ* and *IL-32γ* were detected, but their expression levels were extremely lower than that of *IL-32β*, as well as the lower frequencies were also detected. These results suggested that *IL-32β* was the main isoform in ESCC specimens and EC109 cell line, and might play a key role in the progression of ESCC.

### High expression of IL-32 promotes the migration and invasion of ESCC cell lines

**Fig. 2:**
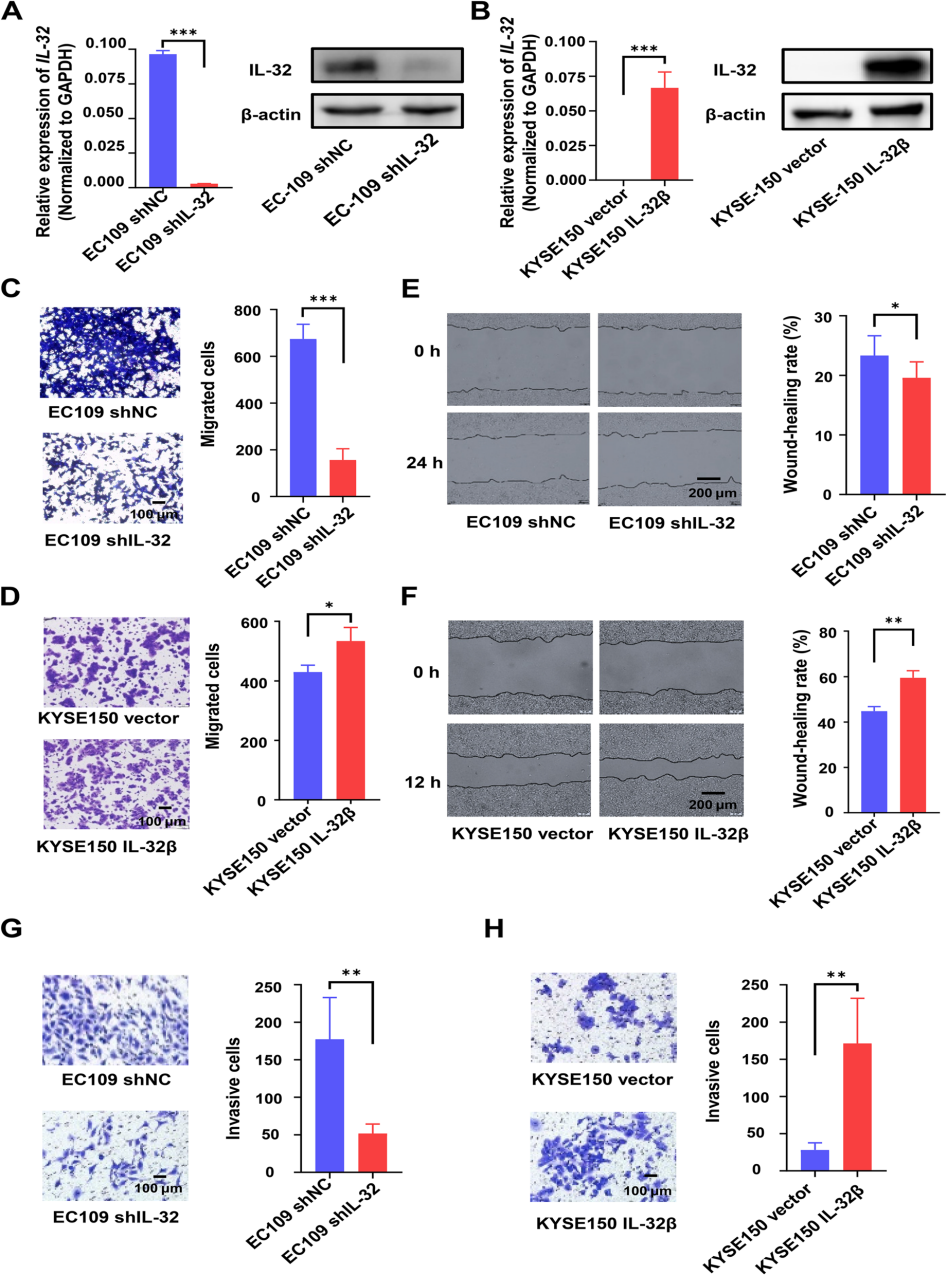
Ectopic overexpression of IL-32 enhances ESCC cells migration and invasion. **A**, **B** The expression level of IL-32 in EC109 shIL-32, EC109 shNC, KYSE150 IL-32β and KYSE150 vector cell lines was detected by qRT-PCR and Western blot assay. Transwell (**C**,** D**), Wound healing (**E**, **F**) and Invasion (**G**, **H**) assays were performed using EC109 shNC, EC109 shIL-32 KYSE150 vector and KYSE150 IL-32β cell lines. Five fields were included per assay, and the experiment was repeated three times. Scale bar, 100 or 200 μm. All data are represented as means ± SEM. Student’s *t*-test, *P < 0.05, **P < 0.01, ***P < 0.001

Clinicopathologic data indicated that overexpression of IL-32 was positively correlated to lymph node metastasis of ESCC, and the main isoform in ESCC and ESCC cell lines was *IL-32β*. Therefore, IL-32β-overexpressing cell line KYSE150 IL-32β and IL-32-knockdown cell line EC109 shIL-32 were established through the lentiviral system. qRT-PCR and Western blot results in Fig. 2A and B showed that these cell lines were successfully established. MTT assay showed that IL-32 had no effects on the growth of these cancer cell lines regardless of overexpression or knockdown (Fig. S4).

Next, transwell assay and wound healing assay were performed to detect the effect of IL-32 on cell migration. Indeed, the migration ability and the wound healing ability of IL-32-knockdown in EC109 cell line (EC109 shIL-32) could be significantly attenuated (Fig. 2C-D). Similarly, the metastasis and the wound healing ability of IL-32β-overexpression KYSE150 cell line (KYSE150 IL-32β) could be significantly enhanced (Fig. 2E-F). The transwell invasion assay with matrigel was performed to assess the role of IL-32 on the invasion ability of ESCC cells. The results suggested that IL-32 promoted the invasive ability of ESCC cell lines (Fig. 2G-H). We also verified the migration of cell lines with low or high expression IL-32. The results showed that IL-32^Low^ cell lines (KYSE150 and KYSE450) have a lower migratory potential than IL-32^High^ cell lines (EC1 and EC109) (Fig. S5). Combined with the results mentioned above, we proposed that IL-32 has no effects on cell growth, but could enhance the migration and invasion of ESCC cells.

### The extracellular secretion of EV-IL-32 rather than soluble form in the supernatant of ESCC cell lines

**Fig. 3:**
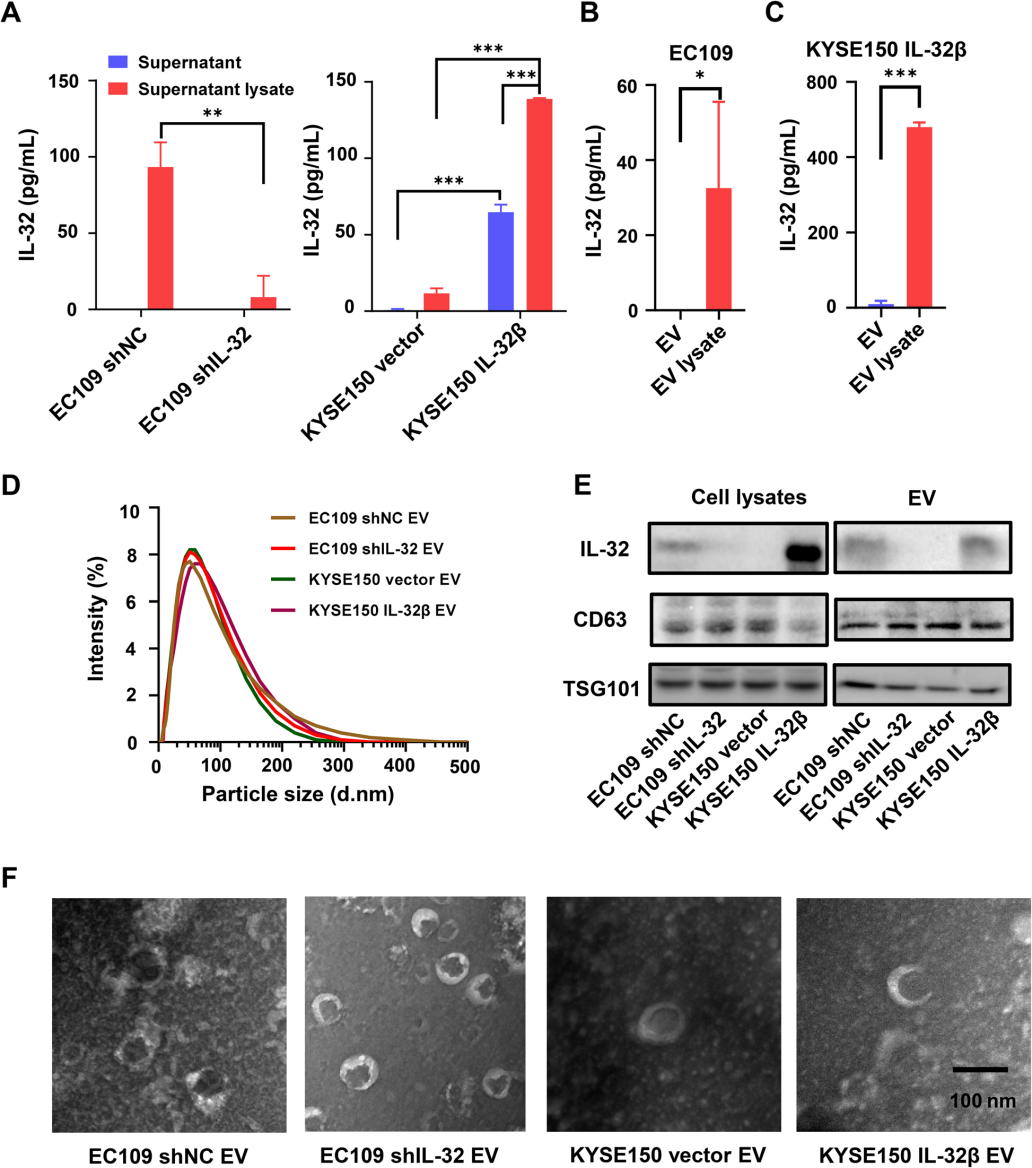
EV-IL-32 was secreted by ESCC cell lines. **A** Cell culture supernatant from EC109 shNC/shIL-32 (Left) and KYSE150 vector/IL-32β (Right) cell lines was treated with or without lysis buffer, and the expression of IL-32 was detected by ELISA. The EV isolated from EC109 (**B**) and KYSE150 IL-32β (**C**) were also treated with or without lysis buffer, and the concentration of IL-32 was measured by ELISA assay. **D** The size distribution of EV isolated from EC109 shNC/shIL-32 and KYSE150 vector/IL-32β cell lines was detected by Malvern spray analyzer. **E** Immunoblotting of EV markers (CD63, TSG101) and IL-32 in EV which were isolated from supernatant of EC109 shNC/shIL-32 and KYSE150 vector/IL-32β cell lines. **F** TEM images of EV purified from EC109 shNC/shIL-32 and KYSE150 vector/IL-32β cell lines. The images showed a mass of round-shaped vesicles. Scale bar = 100 nm. All data are representative of three independent experiments and are represented as means ± SEM. Student’s *t*-test, *P < 0.05, **P < 0.01, ***P < 0.001

As IL-32 could promote the metastasis of ESCC cells, we next explored the mechanisms. We firstly detected the secretion of IL-32 in cultured supernatant of tumor cells (EC109 shNC, EC109 shIL-32, KYSE150 vector, and KYSE150 IL-32β) via ELISA assay. In the culture supernatant of EC109 shNC, the secretion of IL-32 was hardly able to detect. Otherwise in IL-32β overexpression KYSE150 cells, IL-32 was detectable in the supernatant, but the secretion level was significantly upregulated after treated by lysis buffer (Fig. 3A). Our results indicated that IL-32 might be secreted through EV. It has been reported that EV were crucial for the formation of pre-metastatic niche and could contribute to the metastasis of tumor cells [33, 34]. Therefore, we hypothesized that IL-32 may be enclosed in EV to change the tumor microenvironment. To verify the exact secretion of IL-32, we isolated EV by ultra-centrifugation, and treated EV (Derived from EC109 and KYSE150 IL-32β) with or without lysis buffer and detected the secreted IL-32 levels by ELISA assay. As shown in Fig. 3B-C, IL-32 could be detected in the lysate of EV, both endogenous IL-32 expressed cell (EC109) and IL-32β over-expressed cell (KYSE150 IL-32β). Then, the size of EV isolated from cell lines (EC109 shNC, EC109 shIL-32, KYSE150 vector, and KYSE150 IL-32β) were analyzed, and the size was mostly around 30–200 nm (Fig. 3D). Moreover, the EV makers TSG101 and CD63 could be detected in EV, which were confirmed by Western blot (Fig. 3E). Finally, electron microscope analysis showed that the size of isolated EV were scattered from 30 to 200 nm (Fig. 3F). Notably, Western blot assay showed that IL-32 was enriched in the EV isolated from EC109 shNC and KYSE150 IL-32β cells, compared with EC109 shIL-32 and KYSE150 vector cells. Altogether, these results illustrated that the major extracellular secretion of EV-IL-32 was derived from ESCC cells.

### IL-32 expression correlates with the infiltration of M2 macrophage

**Fig. 4:**
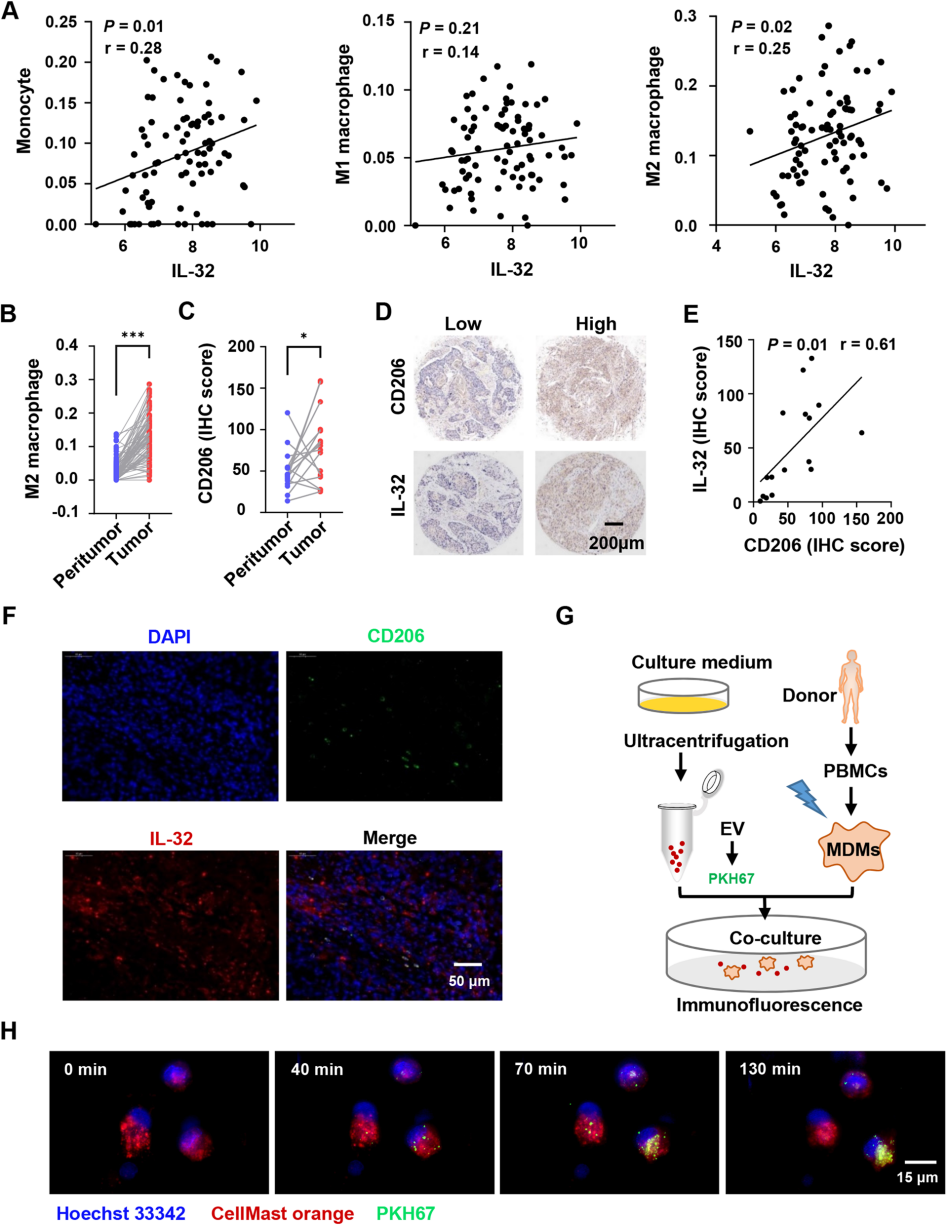
IL-32 expression positively correlates with the infiltration of M2 macrophage in ESCC. **A** Correlation coefficient between *IL-32* and infiltrative monocytes, M1 and M2 macrophages in ESCC tumor tissues, and the subsets of infiltrative cells were performed by the bioinformatic tool xCELL. Each symbol represents an individual patient (*n* = 84). Pearson’s rank correlation test, *P < 0.05. **B** Infiltrative M2 macrophages in ESCC tumor and paired peritumor tissues were analyzed based on RNA microarray (*n* = 84). Paired one-tailed Student’s *t*-test, ***P < 0.001. **C** IHC tissue microarray was used to analyze CD206 expression in ESCC tumor and paired peritumor tissues (*n* = 16). Paired one-tailed Student’s *t*-test, *P < 0.05. **D** Representative IHC images of IL-32 and CD206 in the paired ESCC tumor tissues. Scale bar, 200 μm. **E** Correlation coefficient between IL-32 and CD206 according to IHC scores in ESCC (*n* = 16), Pearson’s rank correlation test, *P < 0.05. **F** Immunofluorescence images of the indicated markers (CD206 and IL-32) in ESCC tumor tissue. Scale bar, 50 μm. **G** Schematic diagram of the EV cocultured with induced MDMs. **H** Confocal fluorescence microscopy was used to detect the process that MDMs (Red) internalized EV (Green) derived from EC109. Scale bar, 15 μm. The data are representative of at least three independent experiments

Aberrant expression of IL-32 has been detected in numerous types of cancer [13–15], but it was poorly understood how EV-IL-32 derived from ESCC cells participated in the metastasis of ESCC. It was well known that the immune suppressive cells in tumor microenvironment could promote the progression of cancer. To explore the correlation between IL-32 and different immune cells, the bioinformatic tool xCELL [35] was used to analyze the subsets of infiltrative immune cells in ESCC tissues (Fig. S6). The results showed that *IL-32* expression levels were positively correlated with the infiltration of monocytes and M2 macrophages (Fig. 4A). Moreover, M2 macrophages were significantly accumulated in ESCC tumor tissues compared with the peritumor tissues (Fig. 4B).

Next, we verified the relationship between IL-32 and M2 macrophages (CD206) in tumor and the paired peritumor tissues by IHC and IF. Our results showed that the expression of CD206 was significantly higher in ESCC tumor tissues (Fig. 4C), which was consistent with the result of RNA microarray. Furthermore, we also detected protein expression levels of IL-32 and CD206 by IHC (Fig. 4D), and the results revealed that the expression level of IL-32 was positively correlated with CD206 (Fig. 4E). We also performed immunofluorescence assay to study whether IL-32 and CD206 were co-expressed. As shown in Fig. 4F and Fig. S7, although most IL-32^+^ cells were tumor cells, there were also a proportion of IL-32^+^CD206^+^ cells in ESCC tissues. These results indicated that IL-32 in ESCC-derived EV might interact with macrophages.

### EV-IL-32 could be internalized into macrophages

To explore whether EV were phagocytosed by macrophages, MDMs (CD14^+^CD11b^+^, Fig. S8) were cocultured with EV derived from EC109. The assay was performed according to the chart showed in Fig. 4G. Malvern spray analyzer was used to determine whether the size was changed after staining with PKH67. Result showed that the same size of EV between staining and unstaining groups (Fig. S9). Confocal fluorescence microscopy was used to confirm the process of EV swallowed by MDMs. The video recorded the process that MDMs phagocytosed the EV labeled with PKH67 (Fig. 4H). An additional movie file shows this in more detail (See Additional movie file 1).

### EV-IL-32 promotes M2 macrophage polarization

**Fig. 5:**
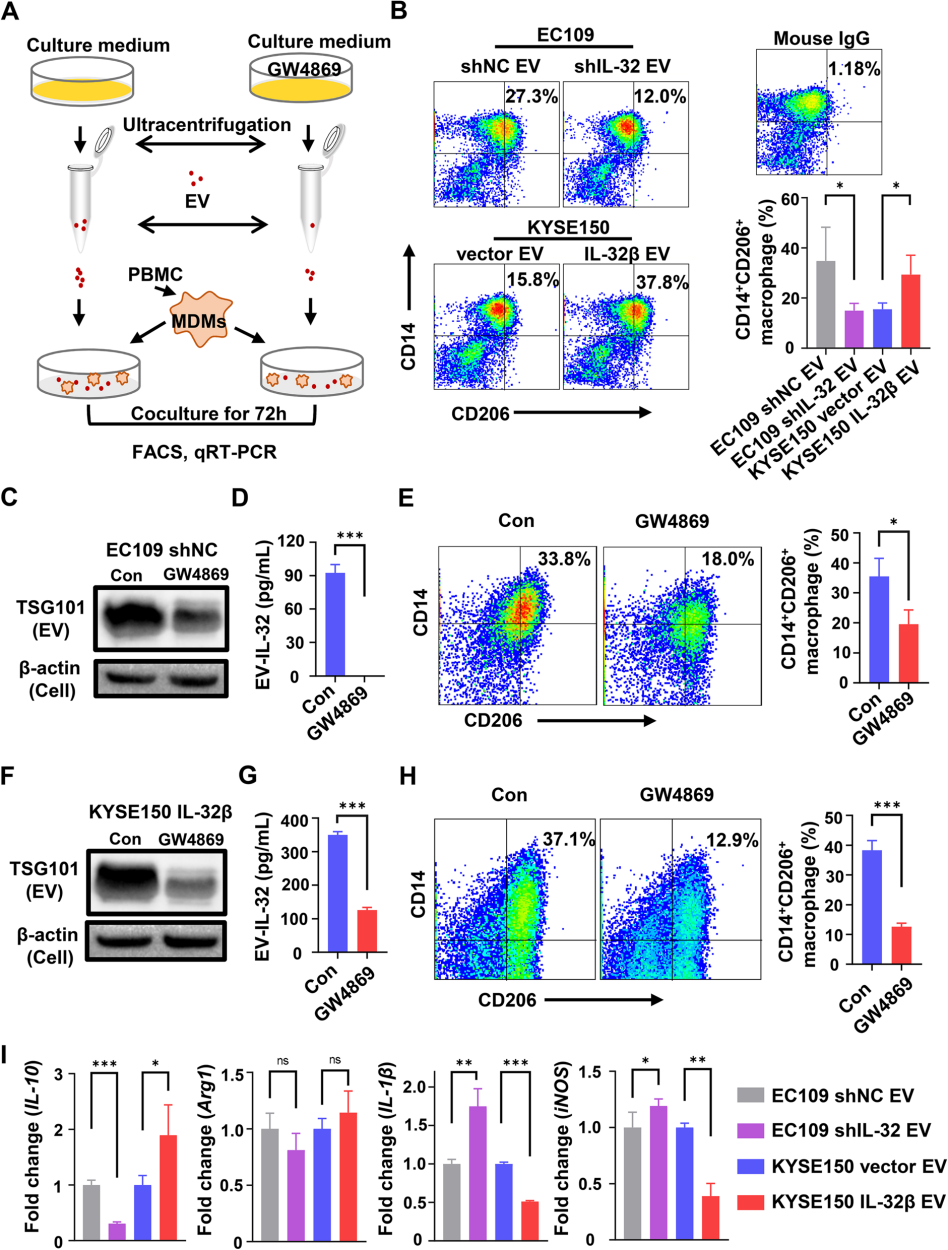
EV-IL-32 promotes macrophage M2 polarization. **A** Schematic chart showed MDMs cocultured with EV treated with or without GW4869. **B** MDMs cocultured with EV (50 µg/mL) isolated from EC109 shNC, EC109 shIL-32, KYSE150 vector and KYSE150 IL-32β for 72 h. The percentage of CD14^+^CD206^+^ macrophages were tested by flow cytometry. **C** The EV were derived from EC109 shNC cells which were treated with or without GW4869 at 10 µM. Western blot analyzed the expression of TSG101 in EV. **D** EV-IL-32 in lysate was detected by ELISA. **E** MDMs cocultured with EV (Derived from 1 × 10^7^ EC109 shNC cells treated with or without GW4869) for 72 h. Flow cytometry analysis of the CD14^+^CD206^+^ macrophages. **F** The EV derived from KYSE150 IL-32β cells treated with or without GW4869 at 10 µM. Western blot analysis of the expression of TSG101 in EV. **G** EV-IL-32 in lysate was detected by ELISA. **H** MDMs cocultured with EV (Derived from 1 × 10^7^ KYSE150 IL-32β cells treated with or without GW4869) for 72 h. Flow cytometry analysis of the CD14^+^CD206^+^ macrophages. **I** MDMs cocultured with EV (50 µg/mL) derived from EC109 shNC, EC109 shIL-32, KYSE150 vector and KYSE150 IL-32β for 48 h. The mRNA expression of M1 (*IL-1β* and *iNOS*) and M2 (*IL-10* and *Arg1*) macrophage associated genes were detected by qRT-PCR. All data are representative of three independent experiments and represented as means ± SEM. Student’s *t*-test, *P < 0.05, **P < 0.01, ***P < 0.001

Since macrophages could phagocytose EV-IL-32, we examined whether their phenotypes and functions could be affected. MDMs were cocultured with EV (50 µg/mL) isolated from ESCC cell lines according to the process in Fig. 5A. The results in Fig. 5B showed that the percentage of M2 macrophages (CD14^+^CD206^+^) were higher in EC109 shNC-derived EV and KYSE150 IL-32β-derived EV groups, compared with EC109 shIL-32-derived EV and KYSE150 vector-derived EV control groups. But the percentage of M1 macrophages (CD14^+^CD80^+^) had no significant difference among each group (Fig. S10).

To further determine whether M2 macrophage polarization was mediated by EV-IL-32, the inhibitor GW4869, which can effectively inhibit the production of EV, was used to treat ESCC cell lines, and the EV derived from each group were determined by western blot assay (Fig. 5C). The IL-32 levels in EV were detected by ELISA. Our results showed that GW4869 significantly inhibited the secretion of EV-IL-32 in EC109 shNC (Fig. 5D). Furthermore, the percentage of M2 macrophages (CD14^+^CD206^+^) significantly reduced when MDMs cocultured with the EV derived from EC109 shNC cells treated with inhibitor (Fig. 5E). Consistent with these results, the secretion of EV-IL-32 in the KYSE150 IL-32β cells treated with GW4869 were also significantly inhibited (Fig. 5F-G), as well as the percentage of M2 macrophages (CD14^+^CD206^+^) significantly reduced (Fig. 5H).

In line with these findings, we also detected the function of macrophage cocultured with tumor cells-derived EV, and qRT-PCR assay was performed to detect the genes expression of cytokines. Our results showed that MDMs cocultured with EV (Derived from EC109 shNC and KYSE150 IL-32β) expressed much higher level of M2 macrophage associated gene *IL-10* and *Arg1* also slightly increased. Conversely, *IL-1β* and *iNOS* genes associated with M1 macrophage were decreased (Fig. 5I). By analyzing GEO dataset, we also discovered that the expression of *IL-32*, *TGF-β* and *IL-10* in ESCC tissues was much higher compared with peritumor tissues (Fig. S11).

To confirm that the effect of EV on macrophages were caused by IL-32, MDMs were treated with recombination IL-32β or M-CSF at 50 ng/mL for 48 h. qRT-PCR assay showed that IL-32β significantly induced the expression of M2 macrophage-related genes (*IL-10* and *Arg1*), and apparently down-regulated M1 macrophage-related genes (*IL-1β*) (Fig. S12). The results suggested that EV-IL-32 could promote the progression of ESCC by M2 macrophage polarization.

### EV-IL-32 derived from ESCC cell promotes M2 macrophage polarization via FAK-STAT3 phosphorylation

**Fig. 6:**
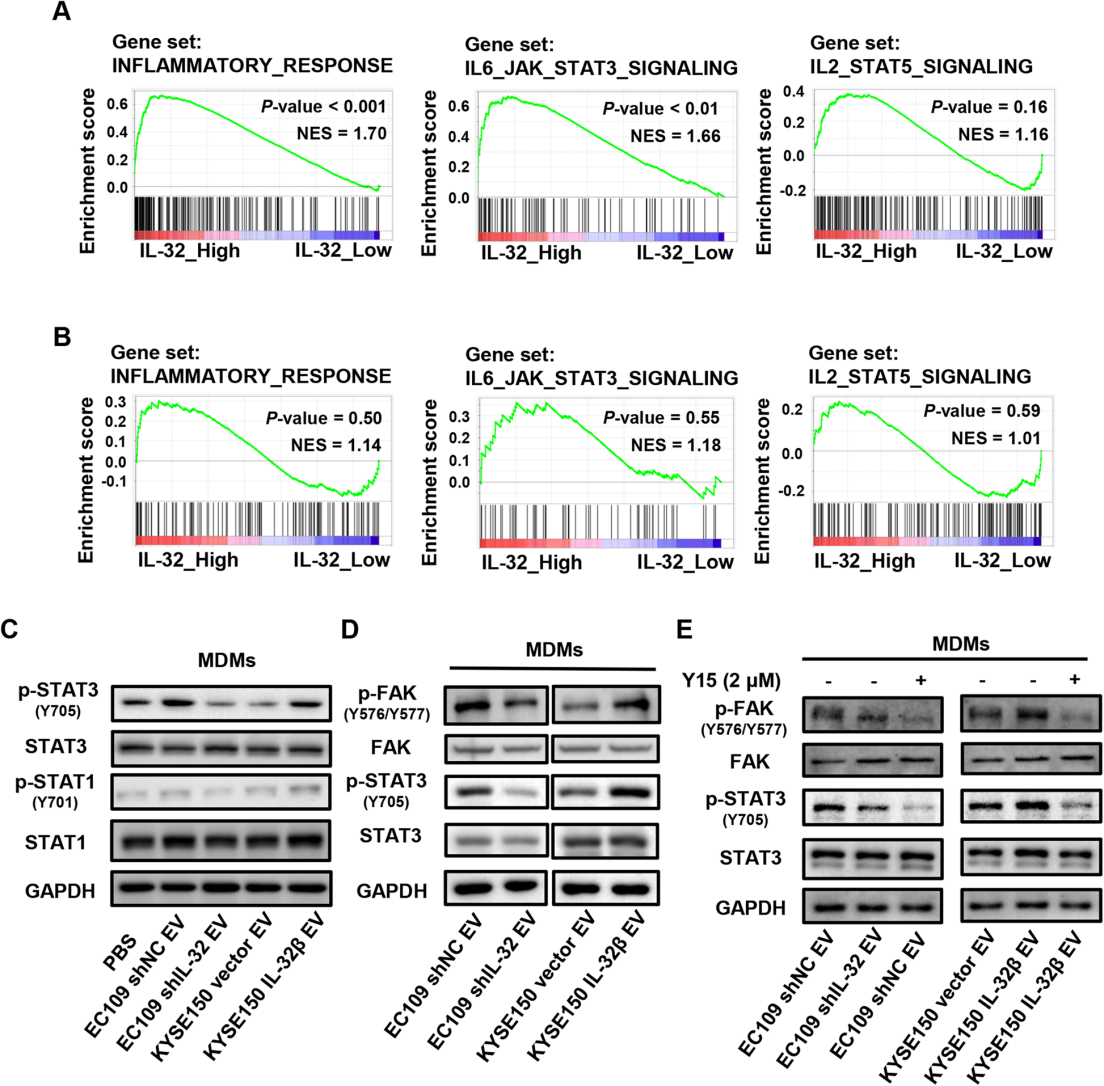
IL-32 in ESCC-derived EV promotes M2 macrophage polarization via FAK-STAT3 activation. **A** Enrichment from GSEA revealed IL-32 expression positively correlated with the inflammatory response and IL-6-JAK-STAT3 signaling pathway in the GSE23400 dataset. **B** The GSEA analysis of EC109 shNC and EC109 shIL-32. **C** Western blot analysis of the expression of STAT1, STAT3, p-STAT1 (Y701) and p-STAT3 (Y705) in MDMs treated with EV. **D** The expression of p-FAK (Y576/Y577) and p-STAT3 (Y705) in MDMs treated with EV were detected by Western blot. **E** EV cocultured with MDMs treated with FAK inhibitor (Y15) at 2 µM for 12 h, and Western blot determined the phosphorylation of FAK and STAT3 in MDMs. Each band is representative of three independent experiments

In order to explore the functions and pathways of IL-32, the expression of IL-32 was analyzed by GSEA with the GSE23400 dataset and EC109 shNC/shIL-32 cells, which revealed the samples of IL-32 highly expressed group were mainly enriched in inflammatory response and IL-6-JAK-STAT3 related pathways (Fig. 6A and B). Moreover, macrophage polarization was related to the activation of STAT1 or STAT3/STAT6 signaling pathway [36, 37]. Therefore, MDMs were cocultured with EV derived from ESCC cells, and western blot assay was performed to investigate the mechanism that IL-32 in ESCC-derived EV facilitated M2 macrophage polarization. Our results showed that the phosphorylation of STAT3 was significantly increased in MDMs treated with EV-IL-32 (EC109 shNC EV and KYSE150 IL-32β EV groups) (Fig. 6C). While, there were no obvious changes in the phosphorylation of STAT1 among different groups. These results demonstrated that EV-IL-32 were internalized by macrophages and promoted M2 macrophage polarization through the phosphorylation of STAT3.

We further sought to find the mechanisms underlying the induction of M2 macrophage polarization by EV-IL-32. FAK, a cytosolic protein, interacted with IL-32, which implied IL-32 may have an important role in cytoplasm [8]. The structure of FAK contains a kinase domain that is responsible for the phosphorylation of Y576/Y577 and FAT domain interacting with paxillin [38]. Because EV-IL-32 could be internalized by MDMs and induced the phosphorylation of STAT3, we next explored whether the IL-32/FAK interaction played an essential role in the phosphorylation of STAT3 during the process. As shown in Fig. 6D, the phosphorylation of FAK and STAT3 were downregulated in MDMs treated with EV derived from EC109 shIL-32, whereas the opposite effect was observed in the group of KYSE150 IL-32β-derived EV. Furthermore, FAK inhibitor (Y15) was used to confirm the hypothesis that FAK regulated by EV-IL-32 can activate the STAT3 in macrophages. Western blot confirmed that the phosphorylation of STAT3 reduced in MDMs when treated with FAK inhibitor (Fig. 6E). These results demonstrated that IL-32 in ESCC-derived EV could promote the phosphorylation of FAK and STAT3 in macrophages, which promoted M2 macrophage polarization.

### IL-32 promotes M2 macrophage polarization in the primary tumor microenvironment

**Fig. 7:**
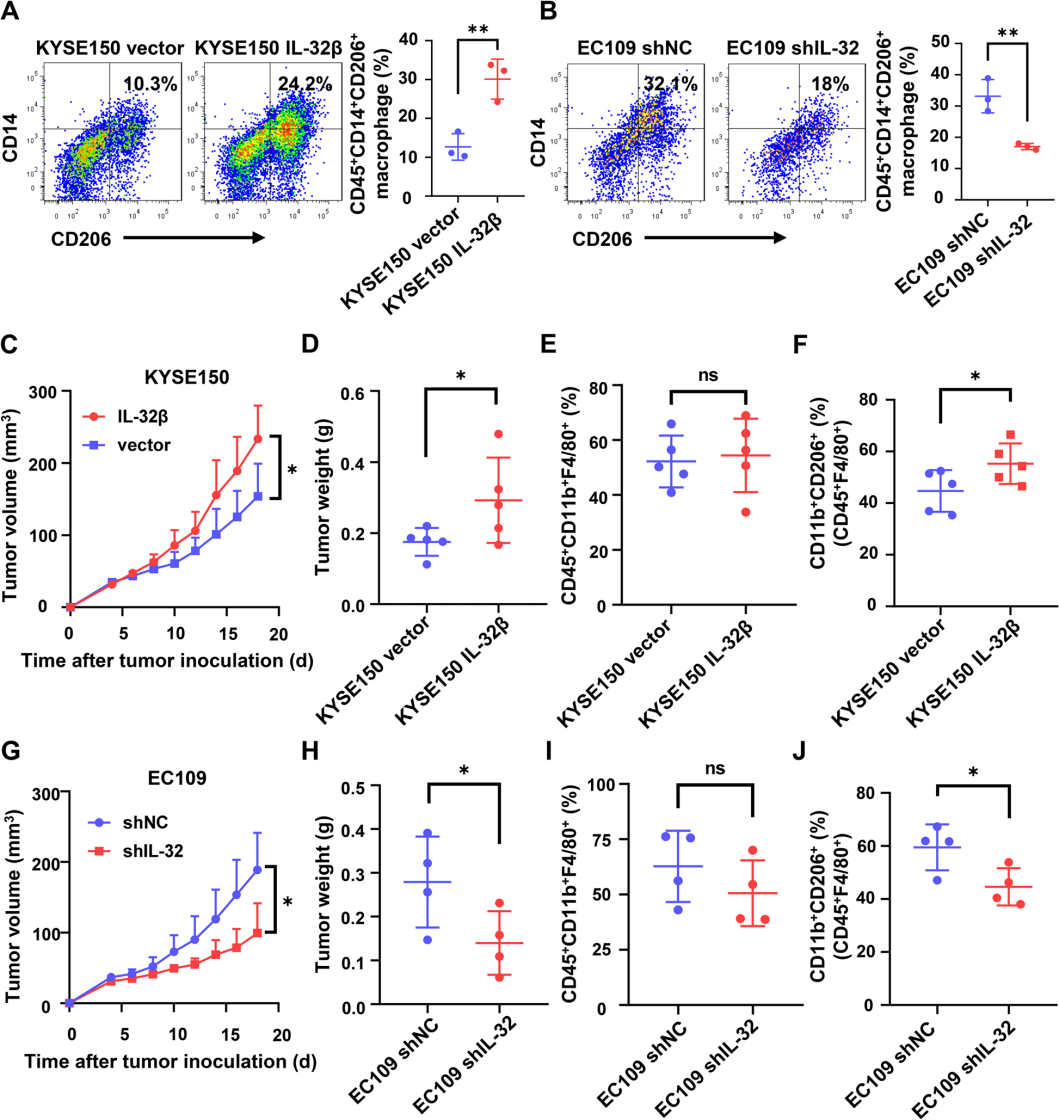
IL-32 promotes M2 macrophage polarization in the primary tumor microenvironment. **A**, **B** Unpolarized MDMs (1 × 10^6^/mouse) and tumor cells (1 × 10^6^/mouse) were subcutaneously injected into nude mice on the right back for 3 days. Phenotypic analysis of M2 macrophages (Anti-human CD45^+^CD14^+^CD206^+^) was performed by flow cytometry. (*n* = 3). **C**, **D** Tumor growth curves and tumor weight showed that IL-32 promoted tumor growth in subcutaneous tumor-bearing mouse model with KYSE150 vector and KYSE150 IL-32β cells (*n* = 5). **E** Intratumoral macrophages were defined as CD45^+^F4/80^+^CD11b^+^ and the proportion was showed (*n* = 5). **F** The percentage of CD206^+^ macrophages was significantly higher in KYSE150 IL-32β group, which was defined as CD45^+^F4/80^+^CD11b^+^CD206^+^ (*n* = 5). **G**, **H** Tumor growth curves and tumor weight showed that knockdown IL-32 inhibited tumor growth in subcutaneous tumor-bearing mouse model with EC109 shNC and EC109 shIL-32 cells (*n* = 4). **I** Intratumoral macrophages were defined as CD45^+^F4/80^+^CD11b^+^ and the proportion was showed (*n* = 4). **J** The percentage of CD206^+^ macrophages was significantly higher in EC109 shNC group, which was defined as CD45^+^F4/80^+^CD11b^+^CD206^+^ (*n* = 4). Data are represented as means ± SEM. Student’s *t*-test, *P < 0.05, **P < 0.01, ***P < 0.001

It is important to show that EV-IL-32 have a function to promote macrophage polarization in vitro. To determine whether IL-32 have a similar role in M2 polarized macrophages in mouse model. We subcutaneously injected MDMs and tumor cells into BALB/c nude mice. Analysis of the phenotype and proportion of M2 macrophages showed that human tumor cells with high IL-32 expression promoted the polarization of M2 macrophages in the primary tumor microenvironment (Fig. 7A-B). Before performing in vivo assay, it is necessary to make sure the cross-reactivity of IL-32 between human and mouse. Previous studies have reported that human IL-32 have cross-reactivity to mouse in athymic nude mice [39]. Further, we verified the biological activity and physiological function of rhIL-32β and EV-IL-32 on bone marrow-derived macrophages (BMDMs) from BALB/c nude mice. The results showed that human rIL-32β and EV-IL-32 could promote the M2 polarization of mouse macrophages (Fig. S13A-C). In addition, rhIL-32β and EV-IL-32 were used to treat mouse BMDMs and the expression of p-FAK and p-STAT3 was detected. Results showed that the phosphorylation of FAK and STAT3 in rhIL-32β or EV-IL-32 treatment group was increased (Fig. S13D), and this activation could be inhibited by FAK inhibitor (Y15) (Fig. S13E). Therefore, we firstly established a subcutaneous ESCC models to investigate the role of IL-32 in the polarization of M2 macrophages in vivo. The results showed that IL-32 was able to promote tumor growth (Fig. 7C-D). Flow cytometry analysis further confirmed macrophage infiltration and macrophage phenotype. Macrophage (F4/80^+^CD11b^+^) infiltration was no obvious change (Fig. 7E). However, the phenotype analysis showed that the ratio of M2 macrophages (CD11b^+^CD206^+^) was remarkably higher in KYSE150 IL-32β group (Fig. 7F and Fig. S14A). The capacity of IL-32 polarizing M2 macrophage was also evaluated in EC109 shNC than EC109 shIL-32 group (Fig. 7G-J and Fig. S14B). The results show that IL-32 have an ability to promote M2 macrophage polarization in the primary tumor microenvironment.

### IL-32 promotes the lung metastasis of ESCC cells in vivo

**Fig. 8:**
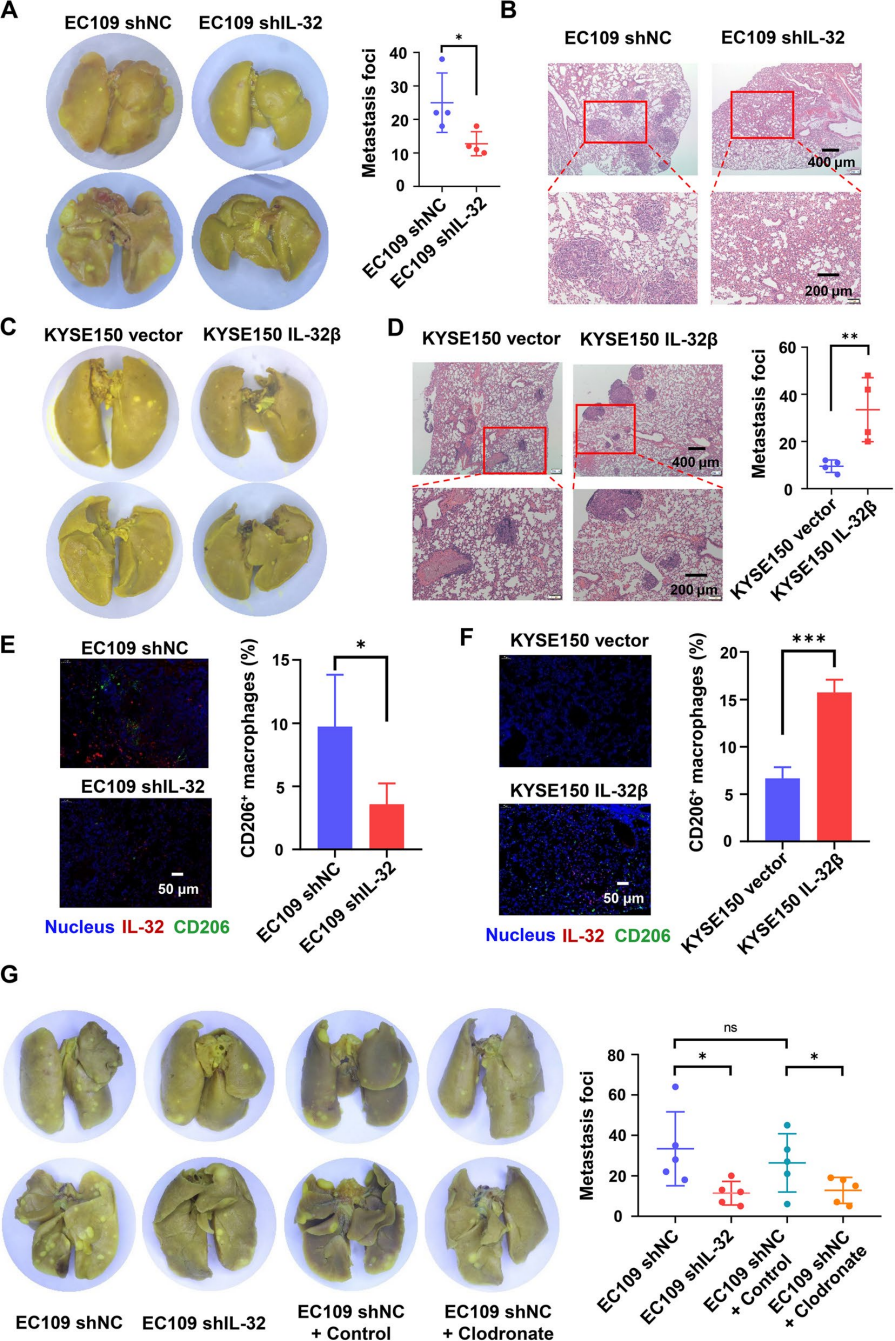
IL-32 promotes lung metastasis in the EC109 and KYSE150 mouse models in vivo. **A** EC109 shIL-32 and EC109 shNC (2 × 10^6^ cells/mouse) were intravenously injected into nude mice. Representative images of excised lungs. Graph showed the number of metastatic foci in the lungs (*n* = 4). **B** Lung metastasis was confirmed by H&E. Scale bars, 400 or 200 μm. **C** KYSE150 IL-32β and KYSE150 vector (1 × 10^6^ cells/mouse) were intravenously injected into nude mice. Representative images of excised lungs were shown. **D** Lung metastasis was confirmed by H&E. Graph showed the number of metastatic foci in the lungs (*n* = 4). Scale bars, 400 or 200 μm. **E**, **F** Immunofluorescence of IL-32 and CD206 in the lungs from mice inoculated with EC109 shIL-32, EC109 shNC, KYSE150 vector and KYSE150 IL-32β cells. Scale bar, 50 μm. **G** BALB/c nude mice were intravenously injected with 100 µL clodronate liposome or control liposome every 4 days after tail vein injection of 2 × 10^6^ EC109 shNC and EC109 shIL-32 cells until 7 weeks. Representative images of excised lungs. Graph showed the number of metastatic foci in the lungs (*n* = 5). Data are represented as means ± SEM. Student’s *t*-test, *P < 0.05, **P < 0.01, ***P < 0.001

Considering that IL-32 could promote the migration of ESCC cells in vitro. We established lung metastasis mouse model to investigate the effects in vivo. Compared with EC-109 shNC group, the lung metastatic nodules both in size and number were significantly decreased in EC109 shIL-32 group, which was confirmed by histological analysis (Fig. 8A-B). These results supported that IL-32 could promote the metastasis of ESCC cells in vivo. Similarly, the histological analysis showed that KYSE150 IL-32β group could also promote the metastasis of ESCC in vivo (Fig. 8C-D).

Since EV-IL-32 could promote the polarization of M2 macrophages in vitro, we next investigated whether the metastatic effected by IL-32 was related to the polarization of M2 macrophages in vivo. Three-color immunofluorescence was performed to analyze the expression of IL-32 and infiltration of M2 macrophages in the lung metastasis model. Compared with EC109 shIL-32 and KYSE150 vector groups, there were more CD206^+^ macrophages in the EC109 shNC and KYSE150 IL-32β groups, respectively (Fig. 8E-F). To confirm M2 macrophages could promote metastasis of ESCC cell lines, EC109 and KYSE150 cell lines were seeded into the upper chamber with 1 × 10^5^/well and EV-IL-32 educated M2 macrophages into the lower chamber with the same density. The results showed that M2 macrophages increased tumor cell mobility and migration (Fig. S15A-C), which are closely related to tumor metastasis.

To further demonstrate whether macrophages promote the metastasis of ESCC cells, macrophages were depleted with Clodronate liposome. The efficiency of macrophage depletion was determined by flow cytometry with analysis of the CD45^+^F4/80^+^CD11b^+^ cells (Fig. S16A). The number of the lung metastatic foci were counted (Fig. 8G), and tumor lesions in the lung tissues was confirmed by H&E staining (Fig. S16B). Metastatic nodules in the lungs were markedly reduced compared with the group of control liposomes. The results showed that depletion of macrophages attenuated the promotion effect of IL-32 on the lung metastasis of EC109 cells. In summary, the promotion effect of IL-32 on the lung metastasis of EC109 cells requires the presence of macrophages.

**Fig. 9:**
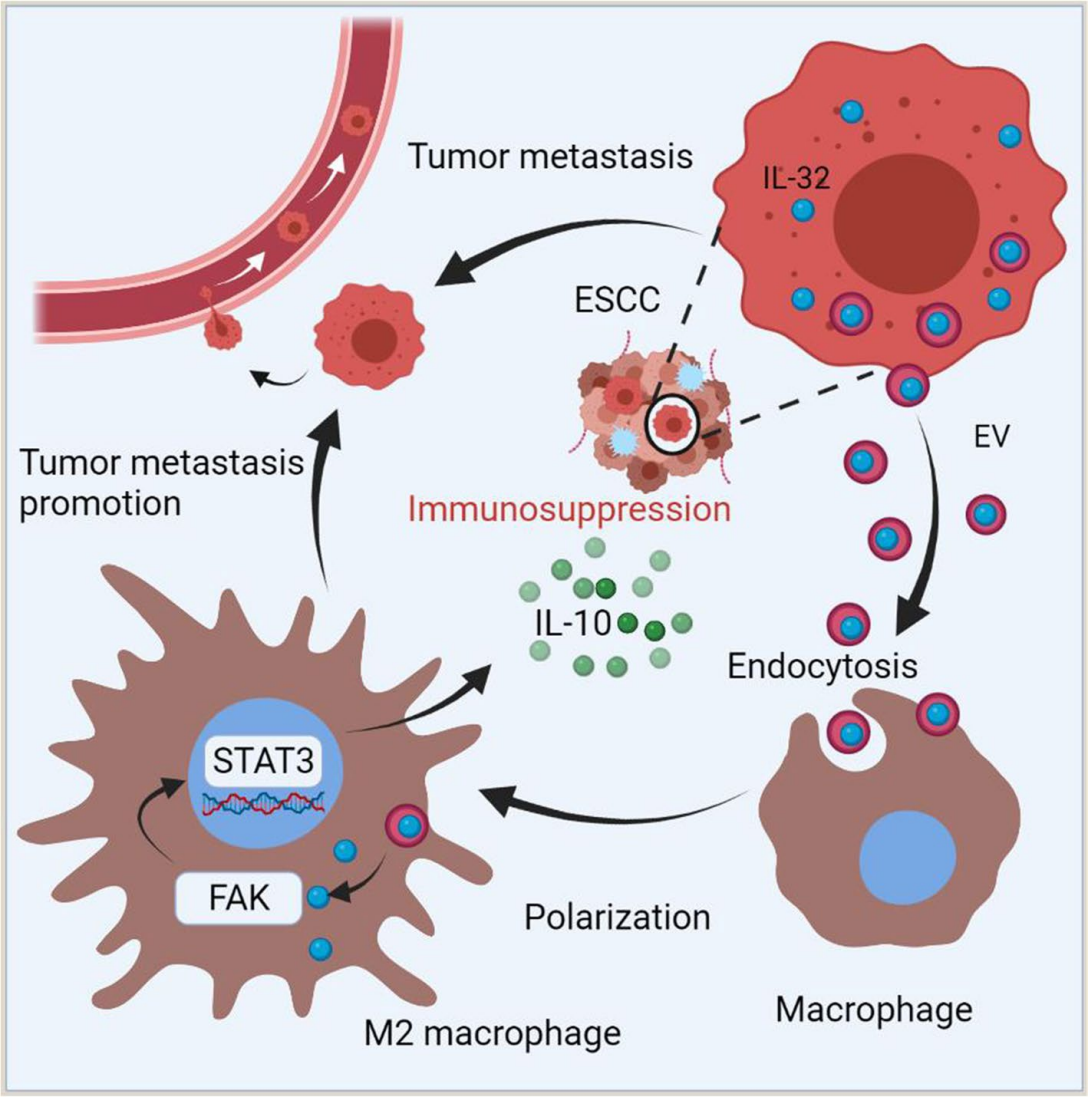
Schematic illustrated that IL-32 in ESCC-derived EV was phagocytosed by macrophage and promoted the polarization of M2 macrophage via p-FAK/p-STAT3 to facilitate the metastasis of ESCC

Overall, these results demonstrated that IL-32 was overexpressed in ESCC and correlated with the poor prognosis of the patients. EV-IL-32 could be phagocytosed by macrophages, and then stimulated the polarization and function of M2 macrophage via FAK/STAT3 pathway, thus promoting the metastasis of ESCC (Fig. 9).

## Discussion

The prognosis of ESCC patients remains poor with five-year survival rate less than 20% [40]. It is urgent to find new therapeutic targets and strategies for the treatment of ESCC.

In the present study, we observed the abnormal overexpression of IL-32 in ESCC by RNA microarray (84 paired ESCC tumor and peritumor tissues) and tissue array immunohistochemistry (56 paired ESCC tumor and peritumor tissues), which was positively associated with lymph node metastasis and poor clinical prognosis in ESCC. As a versatile cytokine, it has been reported that IL-32 has several isoforms, including IL-32α, IL-32β, IL-32γ and IL-32δ. And IL-32α is the mainly isoform which located in intracellular [41]. By sequencing, we verified that *IL-32β* was the main isoform in ESCC tissues and cell lines. The above finding indicated that IL-32β dysregulation may play an important role in the development of ESCC. Importantly, our immunohistochemical results clearly showed that IL-32 was mainly expressed in the cytoplasm of the tumor cell, and was significantly higher in the tumor nest compared with the non-cancerous tissue. Our results suggested that tumor cell was the main source of IL-32. As for the function of IL-32, we found that IL-32 knockdown could attenuate the migration but not the proliferation of ESCC tumor cells in vitro. Therefore, our results suggested that IL-32β was highly expressed in tumor cells and participated in the metastasis of ESCC, and it can be a useful independent biomarker for prognosis.

Previous studies reported that the expression of IL-32 in immune cells can be induced by stimulating factors, such as IFN-γ and TNF-α [42, 43]. Meanwhile, tumor cells are also the main source of IL-32 in the process of neoplasia [13–15]. Curiously, we were hardly able to detect the IL-32 in the culture supernatant of EC109 cells. Otherwise in KYSE150-IL-32β cell, IL-32 was detectable in supernatant, and the secretion level was upregulated after treated by lysis buffer. Thereafter, we verified that the major extracellular secretion form of IL-32 was EV. The presence of cytokines loaded in EV have also been reported in other studies, such as TGFβ1 and CCL2, which could be transferred into target cells and affected their function [44, 45]. Thus, our work indicated that ESCC-derived EV mediated communication and information exchange between cells.

EV are an important mediator for intercellular communication [46]. In recent years, spatial regulation between tumor cells and stromal cells by means of EV to mediate the immune escape, drug resistance and tumorigenesis was a provocative topic [47, 48]. In the present study, we found that IL-32 was positively correlated to monocyte and M2 macrophage as well as co-localization with CD206 in ESCC tumor tissue. The results implied us that EV-IL-32 was phagocytosed by macrophage and mediated the communication between tumor cells and macrophages. Existing published studies reported that recombinant IL-32 promoted M2 polarization [21, 22], but the relationship between EV-IL-32 and macrophage has not been studied before. Studies have reported that tumor cell-derived EV could prepare the distant tumor microenvironment for accelerated metastasis [49, 50]. Unlike the autocrine and paracrine mode of cytokine, EV-IL-32 is internalized into macrophage and play an important role in remote regulation. Accumulated evidences have shown that miRNAs loaded in EV facilitated the polarization of macrophages. Wang et al. reported that miR-301a in tumor-derived EV could mediate M2 macrophage polarization. Park et al. reported that EV derived from hypoxia-induced tumor cells could promote M2-like macrophage polarization [27, 51]. Similarly, our results provided the evidence that the IL-32 in ESCC-derived EV could be phagocytosed by macrophages and regulated the phenotype and function of M2 macrophage. Furthermore, the proportion of M2 macrophage was increased when MDMs co-cultured with EV-IL-32, but this promotion was blocked when GW4869 was used. While the phenotype analysis revealed EV-IL-32 had no effect on M1 macrophage. Above all, our results suggested that EV derived from ESCC cells are more likely to modify local immune microenvironment.

The GSEA was conducted to explore the potential mechanism. The GSEA analysis revealed that the samples overexpressed IL-32 were mainly enriched in inflammatory response and IL-6-JAK-STAT3 related pathways. It was reported that the activation of STAT3 and STAT6 resulted in M2 macrophage polarization, while activation of NF-κB and STAT1 promoted M1 macrophage polarization [36, 52]. It has demonstrated that IL-32-STAT signaling pathway was involved in the regulation of tumor progression in breast cancer [16]. In the coculture assay, we found that EV-IL-32 could promote M2 polarization by STAT3 phosphorylation, and also significantly upregulated the expression of *IL-10*, while downregulated the expression of *IL-1β*. Meanwhile, the polarization of M2 macrophage could further strengthen the immunosuppressive microenvironment.

It has been reported that IL-32 binding to integrin and FAK was essentially for cell migration. FAK, a conserved non-receptor tyrosine kinase, is involved in a variety of biological processes [8, 10, 38, 53]. Gupta et al. found that miR-144/199 inhibited multiple myeloma by downregulating FAK/STAT3 signaling [54, 55]. Furthermore, Shao et al. reported that Curcumin and wikstroflavone B could synergistically suppressed the proliferation and metastasis of nasopharyngeal carcinoma cells via blocking FAK/STAT3 signaling pathway [56]. In our work, IL-32 in ESCC-derived EV promoted the activation of FAK and STAT3 in macrophages, and the phosphorylation of STAT3 reduced when FAK inhibitor was used in macrophage. Ma et al. found that high expression of IL-32 in ESCC cell could enhance the irradiation sensitivity by inhibiting the STAT3 pathway in vitro [12]. Studies have also reported that the patterns of STAT3 signaling pathway were depending on the cell type [57, 58]. In our study, EV-IL-32 can be transported into macrophages to mediate the activation of STAT3 in macrophage. Our results firstly demonstrated that EV-IL-32 had a communication with macrophage and promoted M2 macrophage polarization via FAK-STAT3 pathway in ESCC.

Immunosuppressive microenvironment plays an essential role in the initial stage of metastasis, and leads to the failure of tumor immunotherapy [59, 60]. It has been reported that IL-32 could promote the metastasis of cancer cells by up-regulation the expression of MMP2 and MMP9 [14, 61]. Nold-Petry et al. has shown that IL-32 can also possess its angiogenic property mediated by integrin αVβ3 [62]. We found that IL-32 could regulate the extracellular matrix binding and vascular development (RNA sequence data) (Fig. S17A-D). Moreover, angiogenesis was also observed in subcutaneous tumor model injected with the IL-32 high-expression group (Data were not shown). Angiogenesis may be due to the increased tumor volume and M2 macrophages. In the lung metastasis mouse model, we also found that there were more metastatic nodes (Both in size and number) in IL-32 overexpression group, which was positively correlated with the increased M2 macrophages in tumor microenvironment.

## Conclusions

In summary, we found IL-32 was overexpressed and correlated to the poor prognosis of ESCC. We firstly proved that IL-32 in ESCC-derived EV not soluble form in ESCC cells, and EV-IL-32 could be “eaten” by macrophage and facilitate the M2 polarization via FAK and STAT3 pathway, thus promoting the metastasis of ESCC. These findings illustrated the metastasis promotion mechanism of IL-32 in ESCC, and indicated that IL-32 could serve as a potential therapeutic target in patients with ESCC.

## Supplementary Information

**Additional file 1: Supplemental materials. Figure S1.** IL-32 expression levels in human ESCC tumor and peritumor tissues from GEO cohorts, (A) GSE23400, (B) GSE20347, and (C) GSE45670. Data are represented as mean ± SEM. Student’s *t*-test, *P < 0.05, ***P < 0.001. **Figure S2. ** IL-32 mRNA expression was measured with qRT-PCR in ESCC cell lines and immortalized normal esophageal cell line Het-1A. Data are represented as means ± SEM. **Figure S3.** IL-32β is the main isoform in ESCC tissues and EC109 cell line. (A-B) IL-32 isoforms were confirmed by DNA sequencing in ESCC tissues and EC109 cell line. (C) Summary diagram described the primers distinguish β, γ and θ isoform of IL-32. (D-E) qRT-PCR assay proved IL-32β was the main isoform in ESCC tissues (*n* = 9) and EC109 cell line. Three independent experiments were carried out. Data are represented as means ± SEM. **Figure S4.** MTT assay was performed to measure the growth of EC109 shNC, EC109 shIL-32, KYSE150 vector, and KYSE150 IL-32β cell lines. The data are representative of three independent experiments and represented as means ± SEM. **Figure S5.** The IL-32 expression and migratory ability of ESCC cell lines. The expression level of IL-32 in ESCC cell lines were detected by ELISA (A) and Western blot (B) assays. (C) Transwell assay was performed to detect the migratory potential of ESCC cells, including KYSE150, KYSE450, EC109 and EC1 cell lines. Five fields were included per assay, and the experiment was repeated three times. Scale bar, 100 μm. Data are represented as mean ± SEM. Student’s *t*-test, ***P < 0.001. **Figure S6.** IL-32 and immunocytes in Pearson rank correlation analysis. RNA microarray data was used for analysis. The population of immunocytes were analyzed by xCELL. The expression values of IL-32 were normalized. Pearson’s rank correlation test, *P < 0.05. **Figure S7.** IHC images showed that IL-32 was highly expressed in the cytoplasm of ESCC cells and was significantly higher in the tumor nest compared with the non-cancerous tissue. Scale bar, 50 μm. **Figure S8.** The phenotype of induced MDMs. The subset of MDMs were identified by CD14 and CD11b. **Figure S9.** Malvern spray analyzer showed the size distribution of staining and unstaining EV derived from EC109. **Figure S10.** MDMs cocultured with EV (50 μg/mL) derived from EC109 shNC, EC109 shIL-32, KYSE150 vector and KYSE150 IL-32β for 72 hours. FACS analysis showed the proportion of M1 macrophage determined by CD14 and CD80. The data are representative of three independent experiments and represented as means ± SEM. **Figure S11.** Expression of *IL-32*,*TGFβ* and *IL-10* in ESCC. The expression profile GEO ESCC dataset (GSE75241) was used for analysis (*n* = 15). Data are represented as means ± SEM. Student’s *t*-test, ***P < 0.001. **Figure S12.** MDMs were treated with recombination IL-32β and M-CSF at 50 ng/mL for 48 hours. qRT-PCR was performed to detect the expression of M2 macrophage-related genes (*IL-10, Arg1*) and M1 macrophage-related genes (*IL-1β, iNOS*). All data are representative of three independent experiments and represented as means ± SEM. Student’s *t*-test, *P < 0.05, **P < 0.01, ***P < 0.001. **Figure S13.** Human IL-32 promotes mouse bone marrow-derived macrophages polarized to M2 macrophage. (A) Representative flow plots showing M2 macrophages (CD45^+^F4/80^+^CD11b^+^CD206^+^). (B) BMDMs were induced by 20 ng/mL M-CSF (Peprotech, USA) for 7 days, and were treated with PBS, 50 ng/mL recombinant IL-32β or 50 μg/mL EV-IL-32 for 72 hours. The proportion of M2 macrophage was detected by flow cytometry, and the statistical (C) was shown. (D) Western blot assays for phosphorylated FAK and STAT3 expression using β-actin as control in mouse BMDMs treated with rhIL-32β (50 ng/mL) or EV-IL-32 (50 μg/mL) for 3 hours. (E) FAK inhibitor (Y15, 5 μM) decreased the phosphorylation of STAT3 in BMDMs treated rhIL-32β or EV-IL-32. The figures are representatives of three replicates. Scale bar, 100 μm. Student’s *t*-test, **P < 0.01, ***P < 0.001. **Figure S14.** Representative dot plots of intratumoral M2 macrophages were defined as CD45^+^F4/80^+^CD11b^+^cells and the proportion was showed in mouse model of KYSE150 vector, KYSE150 IL-32β (A), EC109 shNC and EC109 shIL-32 (B). **Figure S15.** EV-IL-32 educated M2 macrophage promotes metastasis of EC109 and KYSE150. (A) The coculture system proved that EV-IL-32 induced M2 macrophages promoted migration of ESCC cells in vitro. (B-C) EC109 and KYSE150 were seeded into the upper chamber with 1×10^5^/well and EV-IL-32 educated M2 macrophages into the lower chamber with the same density. Five fields were included per assay, and the experiment was repeated three times. Scale bar, 100 μm. Data are represented as means ± SEM. Student’s *t*-test, *P < 0.05, ***P < 0.001. **Figure S16.** Depletion of macrophages attenuating EC109 cells lung metastasis in vivo. (A) The percentage of F4/80^+^CD11b^+^ macrophages from blood were analyzed the day after administration of Control liposome or Clodronate liposome using flow cytometry. (B) Lung metastasis was confirmed by H&E (*n* = 4). Scale bars, 400 or 200 μm. Data are represented as means ± SEM. Student’s *t*-test, *P < 0.05, **P < 0.01. **Figure S17.** The global transcriptional profile of EC109 shNC and EC109 shIL-32 was assessed by RNA-Seq. (A) The GO enrichment revealed that IL-32 knockdown in EC109 mainly affected vascular system development and extracellular matrix binding. (B) The data from RNA-seq was used to analyze the up-regulation and down-regulation genes in extracellular matrix binding. (C-D) qRT-RCR was performed to determine the fold change of genes about extracellular matrix binding in EC109 shNC, EC109 shIL-32, KYSE150 vector and KYSE150 IL-32β. Data are represented as means ± SEM. Student’s *t*-test, *P < 0.05, **P< 0.01, ***P < 0.001. **Table S1.** Primers for qRT-PCR to distinguish each isoform of IL-32. **Table S2.** Primers for qRT-PCR to amplify genes. **Table S3.** Primary antibodies for FACS. **Table S4.** Primary antibodies for western blot.

**Additional file 2: Additional movie file 1.** The process of EVs swallowed by MDMs.

## Data Availability

The data generated and analyzed during this study are included in this manuscript and its supplementary information files.

## Abbreviations

ESCC: Esophageal squamous cell carcinoma
IL-32: Interleukin-32
EV: Extracellular vesicle
FAK: Focal adhesion kinase 1
STAT3: Signal transducer and activator of transcription 3
NF-κB: Nuclear factor kappa-B
MAPK: Mitogen-activated protein kinase
IHC: Immunohistochemistry
NC: Normal control
miRNA: MicroRNA
lncRNA: Long non-coding RNA
qRT-PCR: Real-time quantitative reverse transcription polymerase chain reaction
PBMCs: Peripheral blood mononuclear cells
MDMs: Monocyte-derived macrophages
shRNA: Short hairpin RNA
ELISA: Enzyme-linked immunosorbent assay
IF: Immunofluorescence
TEM: Transmission electron microscope
GSEA: Gene Set Enrichment Analysis
GEO: Gene Expression Omnibus
