# Supplementary material for: Extracellular vesicle IL-32 promotes the M2 macrophage polarization and metastasis of esophageal squamous cell carcinoma via FAK/STAT3 pathway
Source: J Exp Clin Cancer Res. 2022 Apr 15;41:145. doi: 10.1186/s13046-022-02348-8 (PMC9013041; doi:10.1186/s13046-022-02348-8)
Supplement: Supplementary file 1 — Additional file 1: Supplemental materials. Figure S1. IL-32 expression levels in human ESCC tumor and peritumor tissues from GEO cohorts, (A) GSE23400, (B) GSE20347, and (C) GSE45670. Data are represented as mean ± SEM. Student’s t-test, *P < 0.05, ***P < 0.001. Figure S2. IL-32 mRNA expression was measured with qRT-PCR in ESCC cell lines and immortalized normal esophageal cell line Het-1A. Data are represented as means ± SEM. Figure S3. IL-32β is the main isoform in ESCC tissues and EC109 cell line. (A-B) IL-32 isoforms were confirmed by DNA sequencing in ESCC tissues and EC109 cell line. (C) Summary diagram described the primers distinguish β, γ and θ isoform of IL-32. (D-E) qRT-PCR assay proved IL-32β was the main isoform in ESCC tissues (n = 9) and EC109 cell line. Three independent experiments were carried out. Data are represented as means ± SEM. Figure S4. MTT assay was performed to measure the growth of EC109 shNC, EC109 shIL-32, KYSE150 vector, and KYSE150 IL-32β cell lines. The data are representative of three independent experiments and represented as means ± SEM. Figure S5. The IL-32 expression and migratory ability of ESCC cell lines. The expression level of IL-32 in ESCC cell lines were detected by ELISA (A) and Western blot (B) assays. (C) Transwell assay was performed to detect the migratory potential of ESCC cells, including KYSE150, KYSE450, EC109 and EC1 cell lines. Five fields were included per assay, and the experiment was repeated three times. Scale bar, 100 μm. Data are represented as mean ± SEM. Student’s t-test, ***P < 0.001. Figure S6. IL-32 and immunocytes in Pearson rank correlation analysis. RNA microarray data was used for analysis. The population of immunocytes were analyzed by xCELL. The expression values of IL-32 were normalized. Pearson’s rank correlation test, *P < 0.05. Figure S7. IHC images showed that IL-32 was highly expressed in the cytoplasm of ESCC cells and was significantly higher in the tumor nest compared with the non-ca [file 13046_2022_2348_MOESM1_ESM.docx]

**Supplementary information**


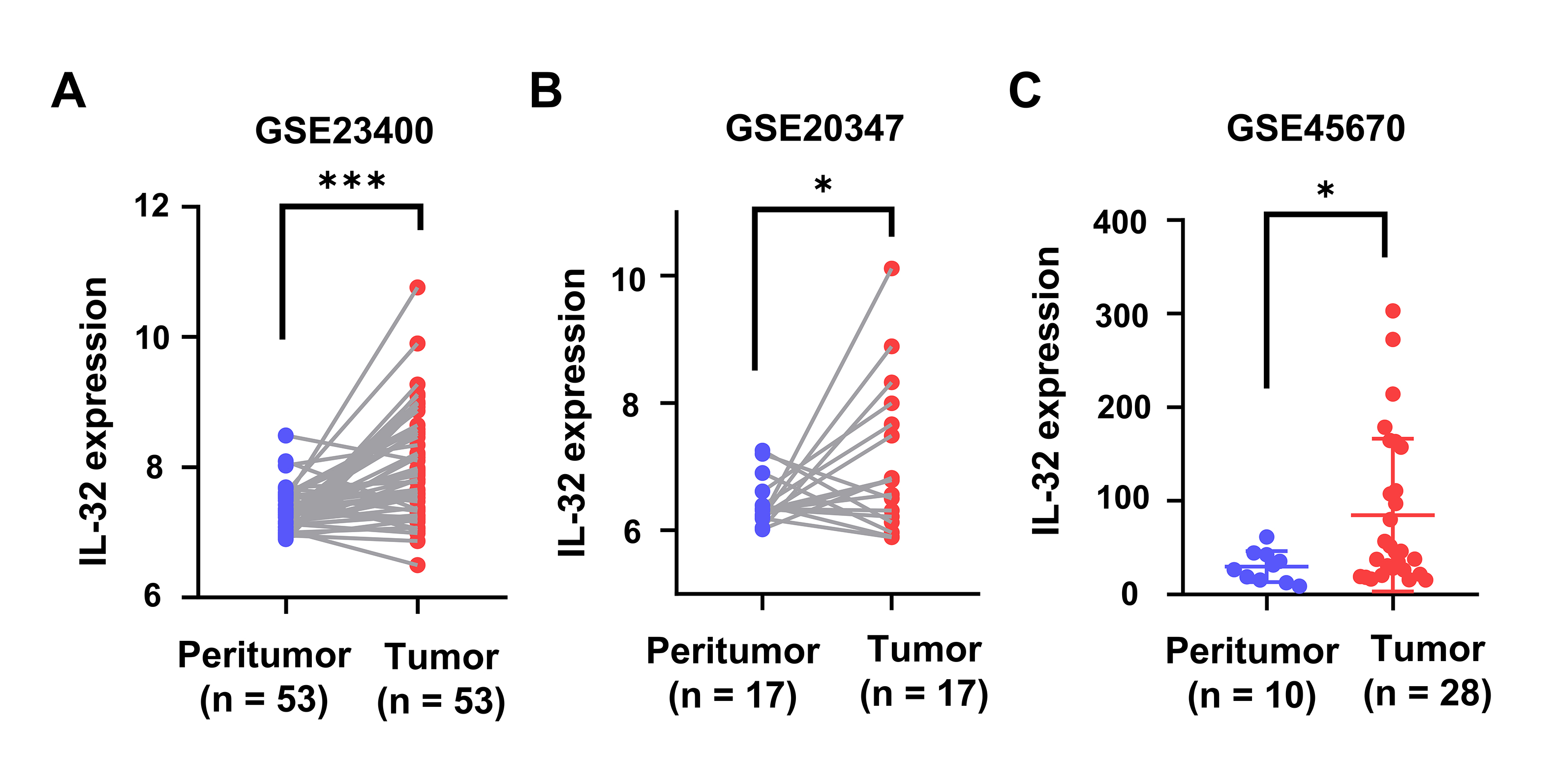


**Figure S1** IL-32 expression levels in human ESCC tumor and peritumor tissues from GEO cohorts, (A) GSE23400, (B) GSE20347, and (C) GSE45670. Data are represented as mean ± SEM. Student’s *t*-test, **P* < 0.05, ****P* < 0.001.


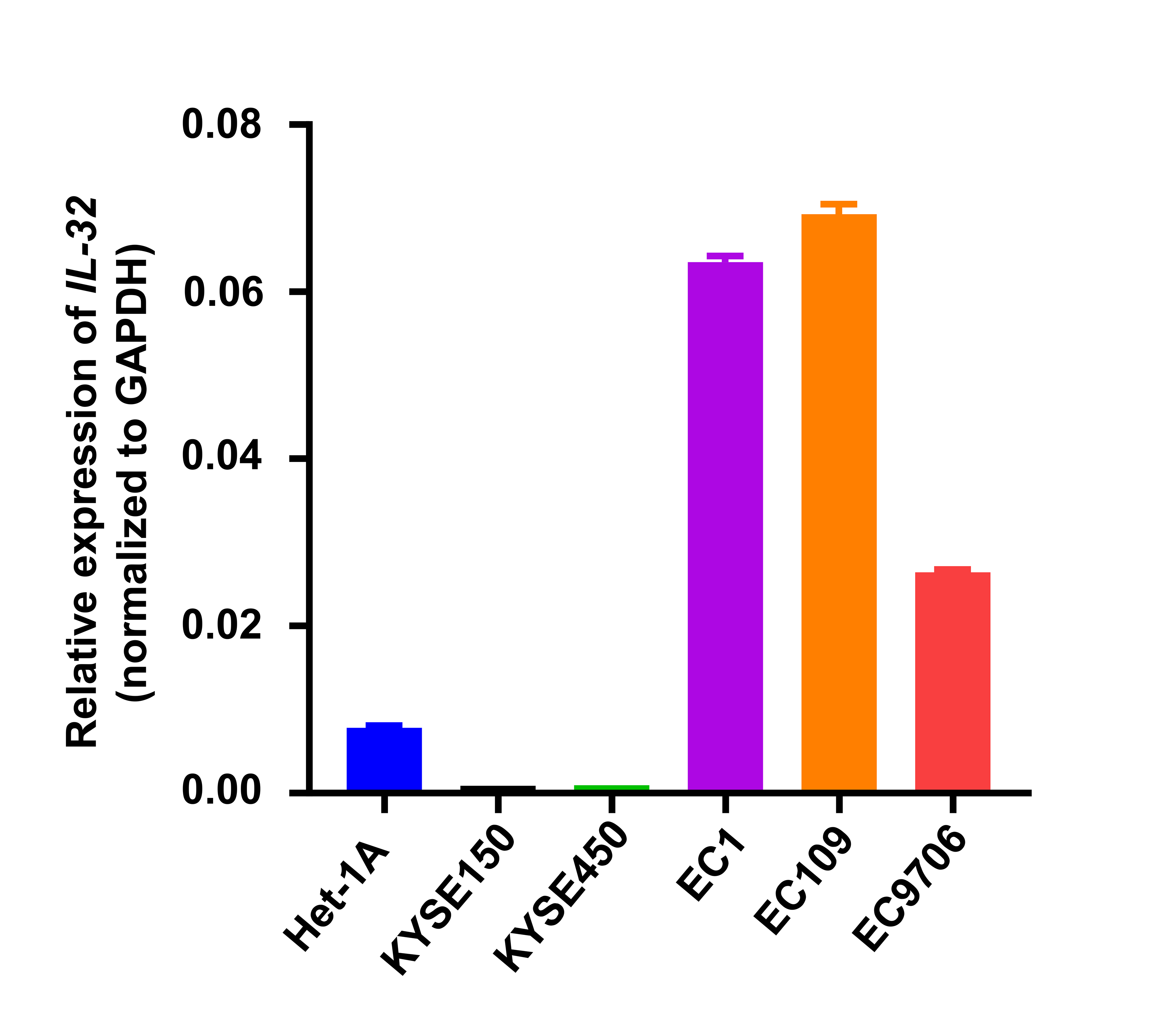


**Figure S2** IL-32 mRNA expression was measured with qRT-PCR in ESCC cell lines and immortalized normal esophageal cell line Het-1A. Data are represented as means ± SEM.


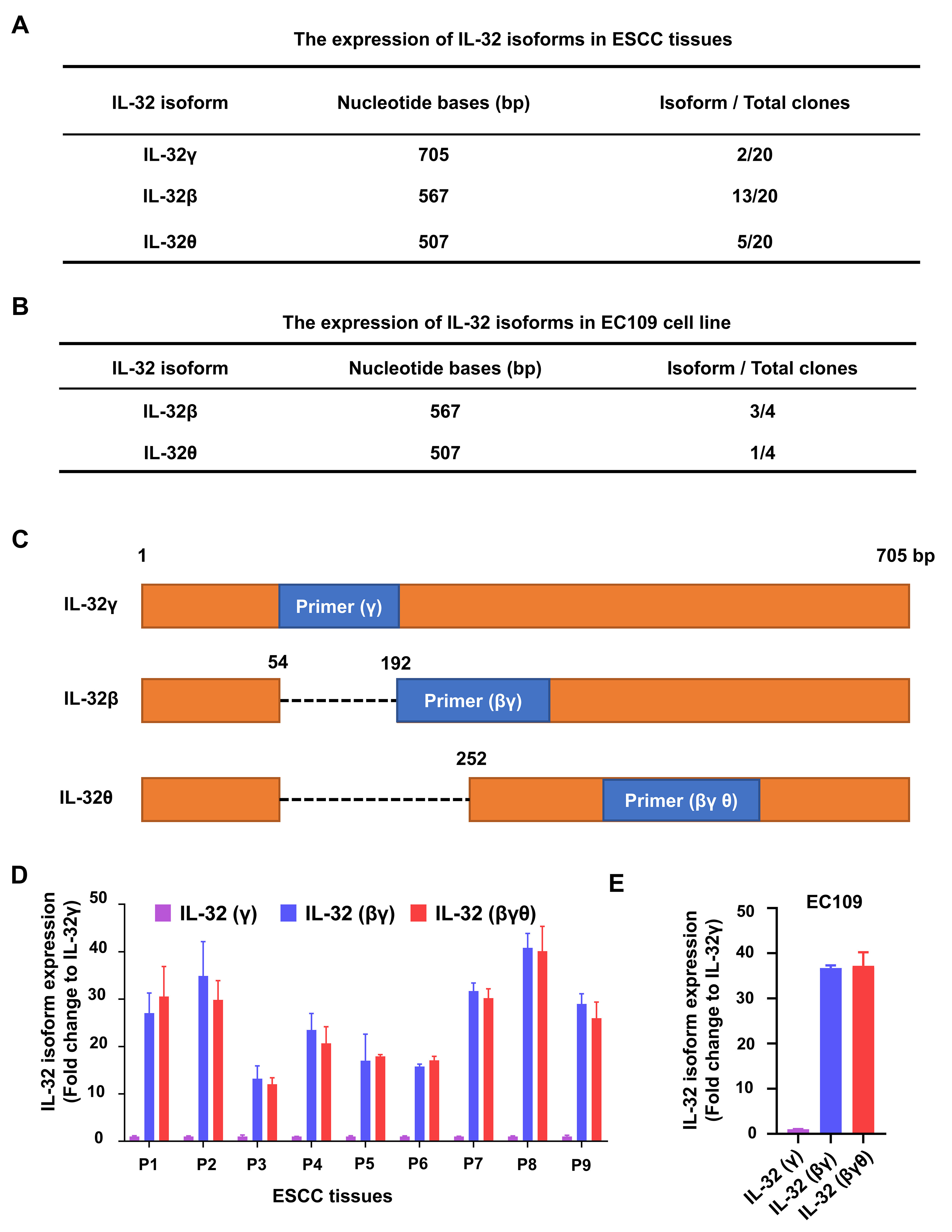


**Figure S3** IL-32β is the main isoform in ESCC tissues and EC109 cell line. (A-B) IL-32 isoforms were confirmed by DNA sequencing in ESCC tissues and EC109 cell line. (C) Summary diagram described the primers distinguish β, γ and θ isoform of IL-32. (D-E) qRT-PCR assay proved IL-32β was the main isoform in ESCC tissues (n = 9) and EC109 cell line. Three independent experiments were carried out. Data are represented as means ± SEM.


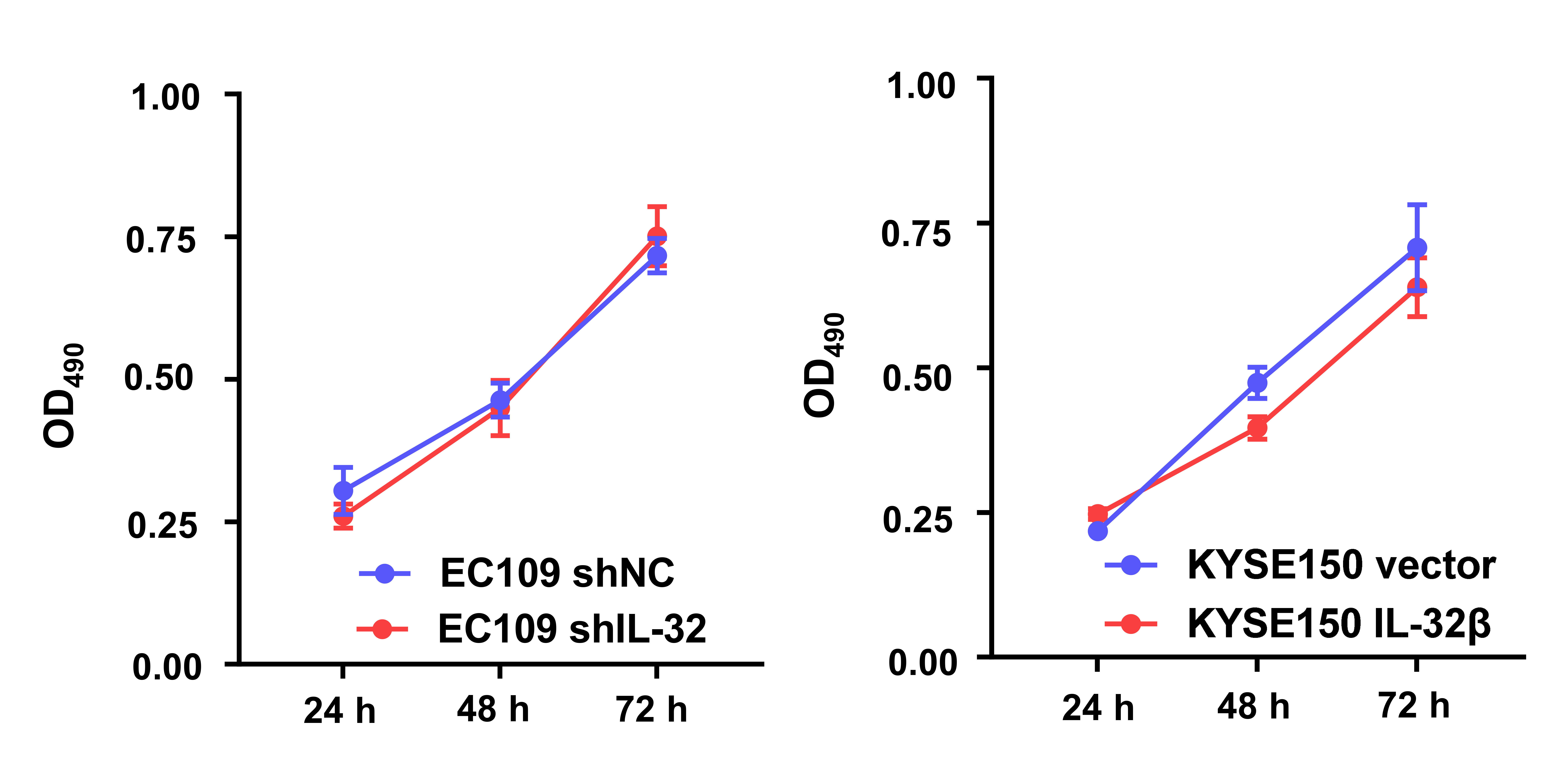


**Figure S4** MTT assay was performed to measure the growth of EC109 shNC, EC109 shIL-32, KYSE150 vector, and KYSE150 IL-32β cell lines. The data are representative of three independent experiments and represented as means ± SEM.


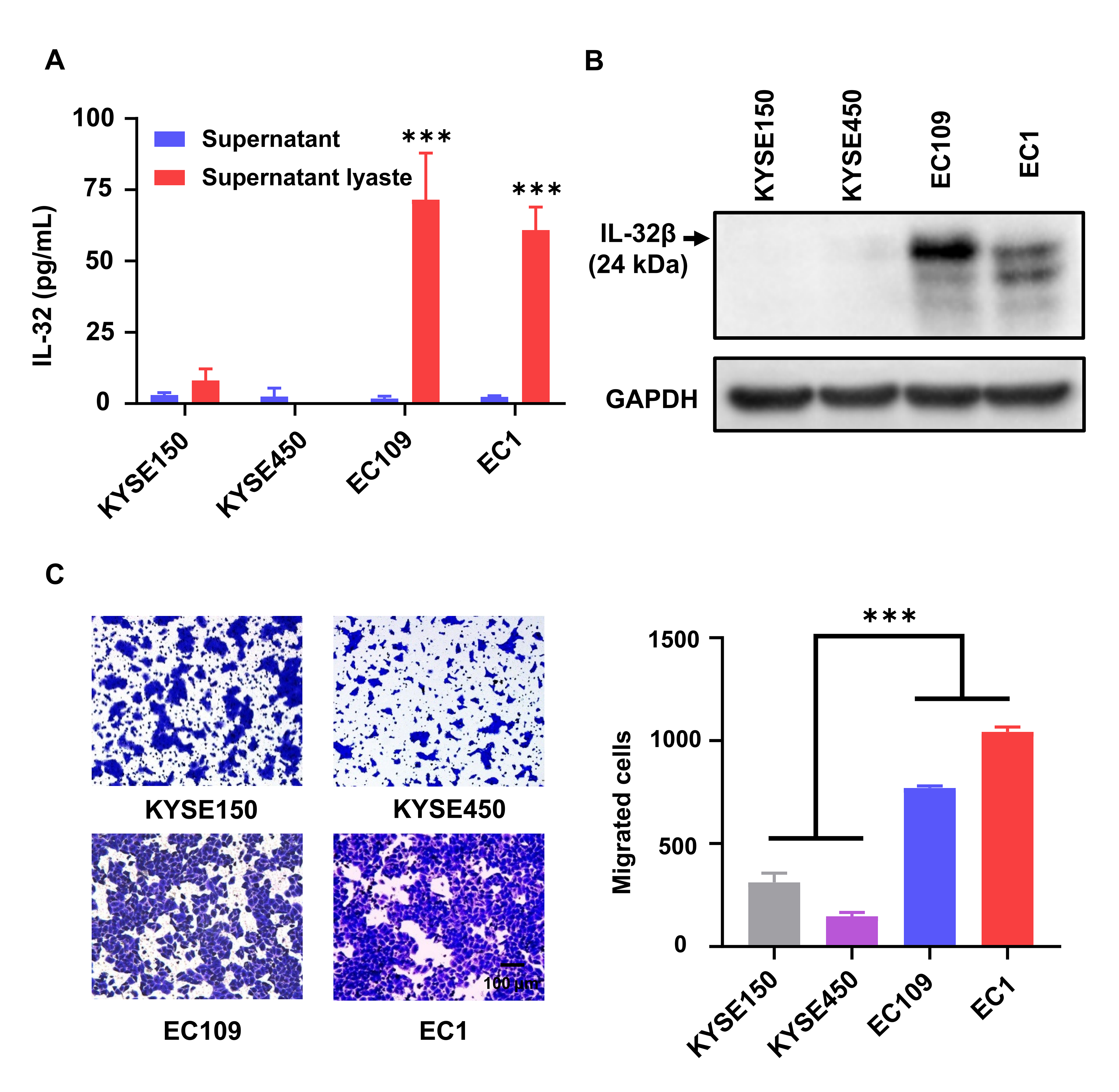


**Figure S5** The IL-32 expression and migratory ability of ESCC cell lines. The expression level of IL-32 in ESCC cell lines were detected by ELISA (A) and Western blot (B) assays. (C) Transwell assay was performed to detect the migratory potential of ESCC cells, including KYSE150, KYSE450, EC109 and EC1 cell lines. Five fields were included per assay, and the experiment was repeated three times. Scale bar, 100 μm. Data are represented as mean ± SEM. Student’s *t*-test, ****P* < 0.001.


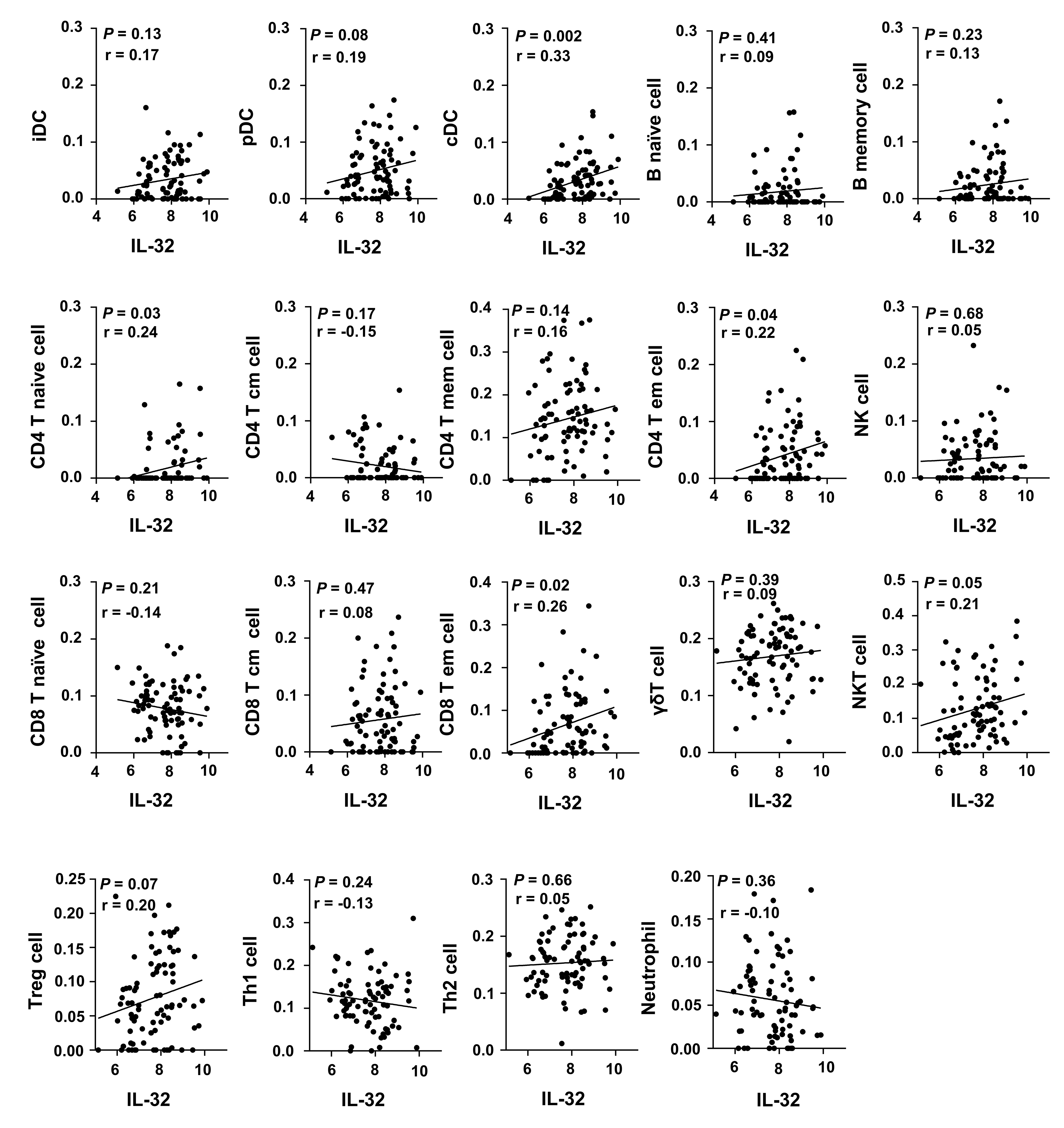


**Figure S6** IL-32 and immunocytes in Pearson rank correlation analysis. RNA microarray data was used for analysis. The population of immunocytes were analyzed by xCELL. The expression values of IL-32 were normalized. Pearson’s rank correlation test, **P* < 0.05.


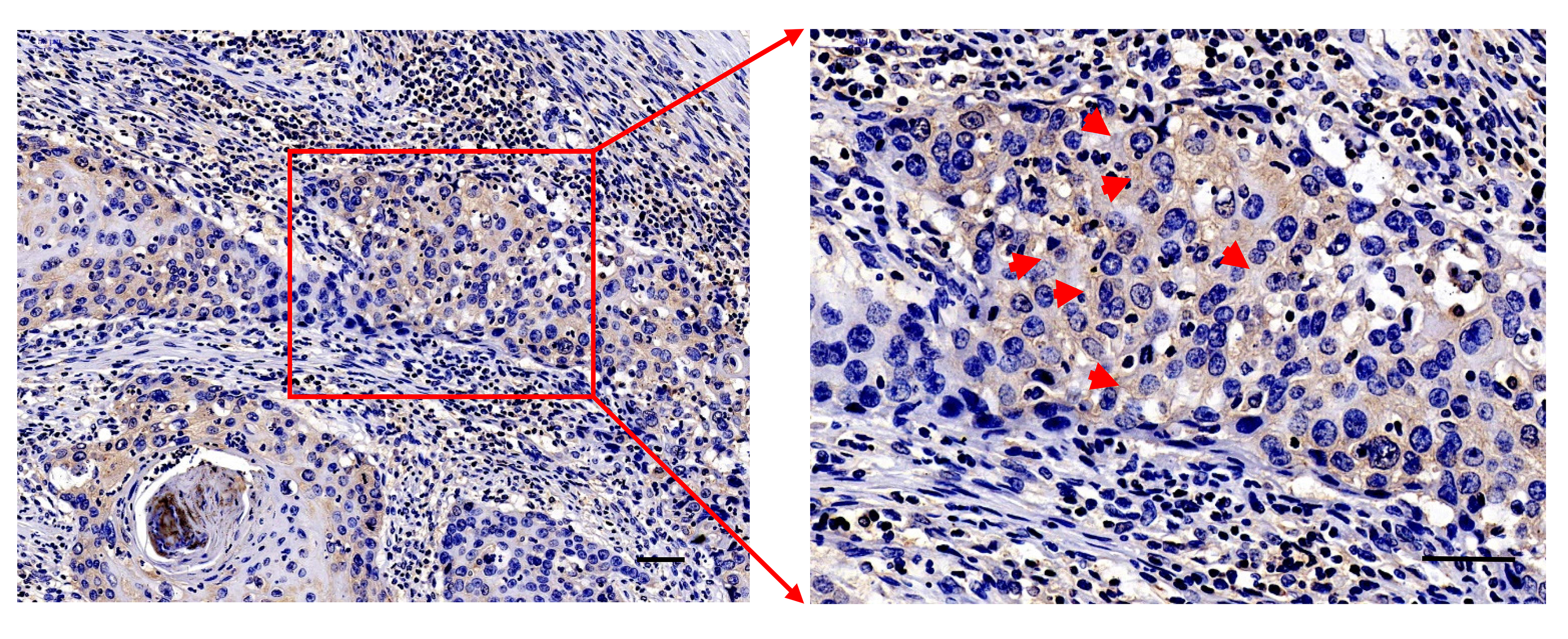


**Figure S7** IHC images showed that IL-32 was highly expressed in the cytoplasm of ESCC cells and was significantly higher in the tumor nest compared with the non-cancerous tissue. Scale bar, 50 μm.


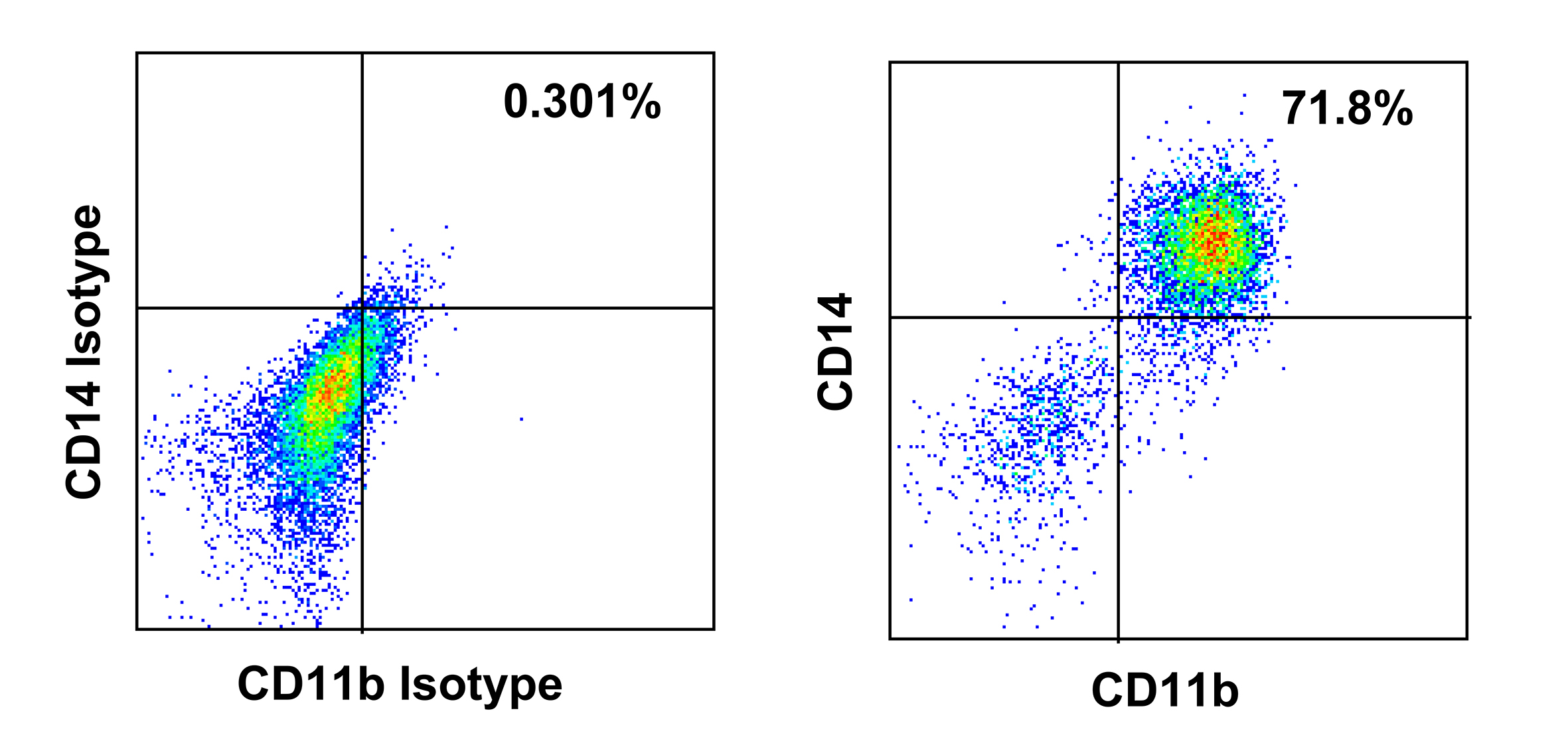


**Figure S8** The phenotype of induced MDMs. The subset of MDMs were identified by CD14 and CD11b.


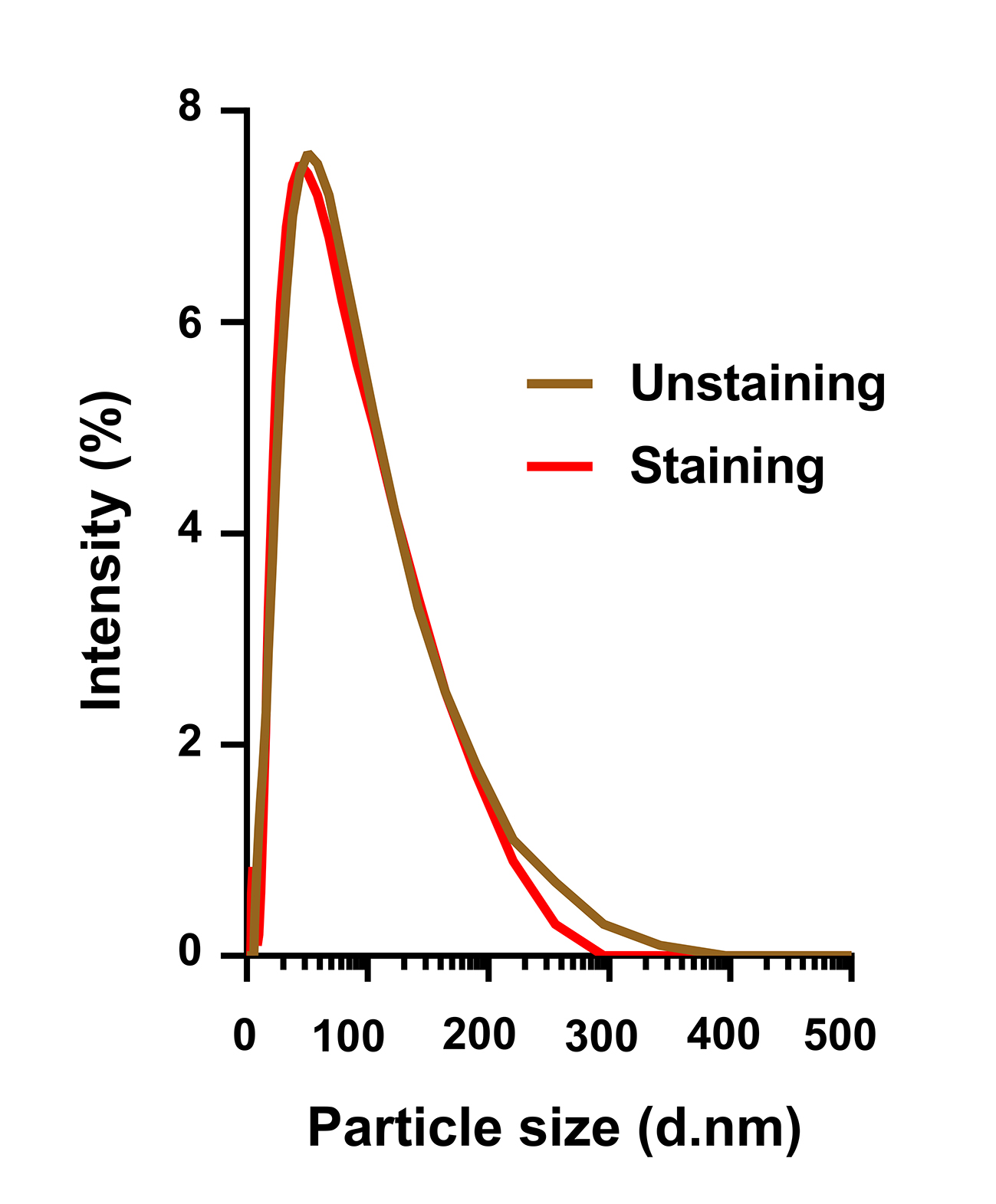


**Figure S9** Malvern spray analyzer showed the size distribution of staining and unstaining EV derived from EC109.


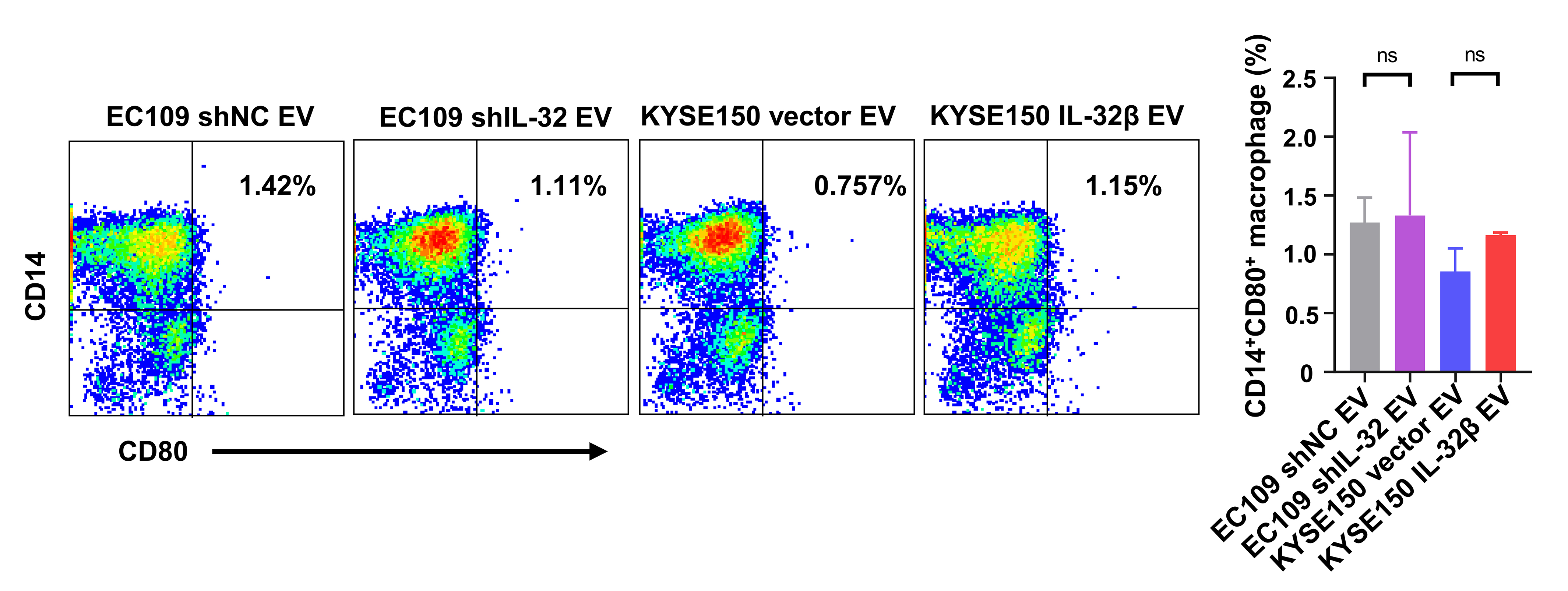


**Figure S10** MDMs cocultured with EV (50 μg/mL) derived from EC109 shNC, EC109 shIL-32, KYSE150 vector and KYSE150 IL-32β for 72 hours. FACS analysis showed the proportion of M1 macrophage determined by CD14 and CD80. The data are representative of three independent experiments and represented as means ± SEM.


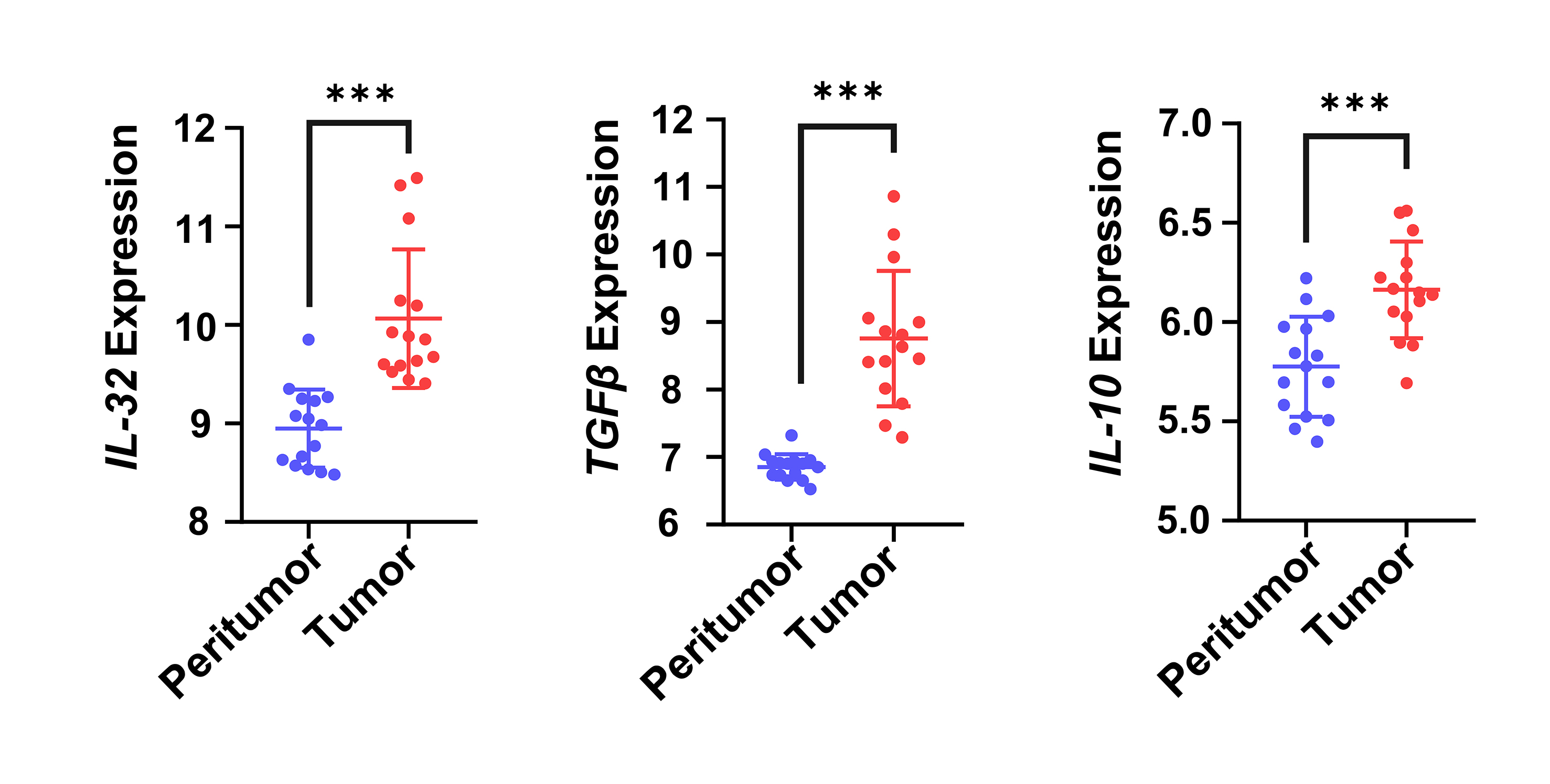


**Figure S11** Expression of *IL-32*, *TGFβ* and *IL-10* in ESCC. The expression profile GEO ESCC dataset (GSE75241) was used for analysis (n = 15). Data are represented as means ± SEM. Student’s *t*-test, ****P* < 0.001.


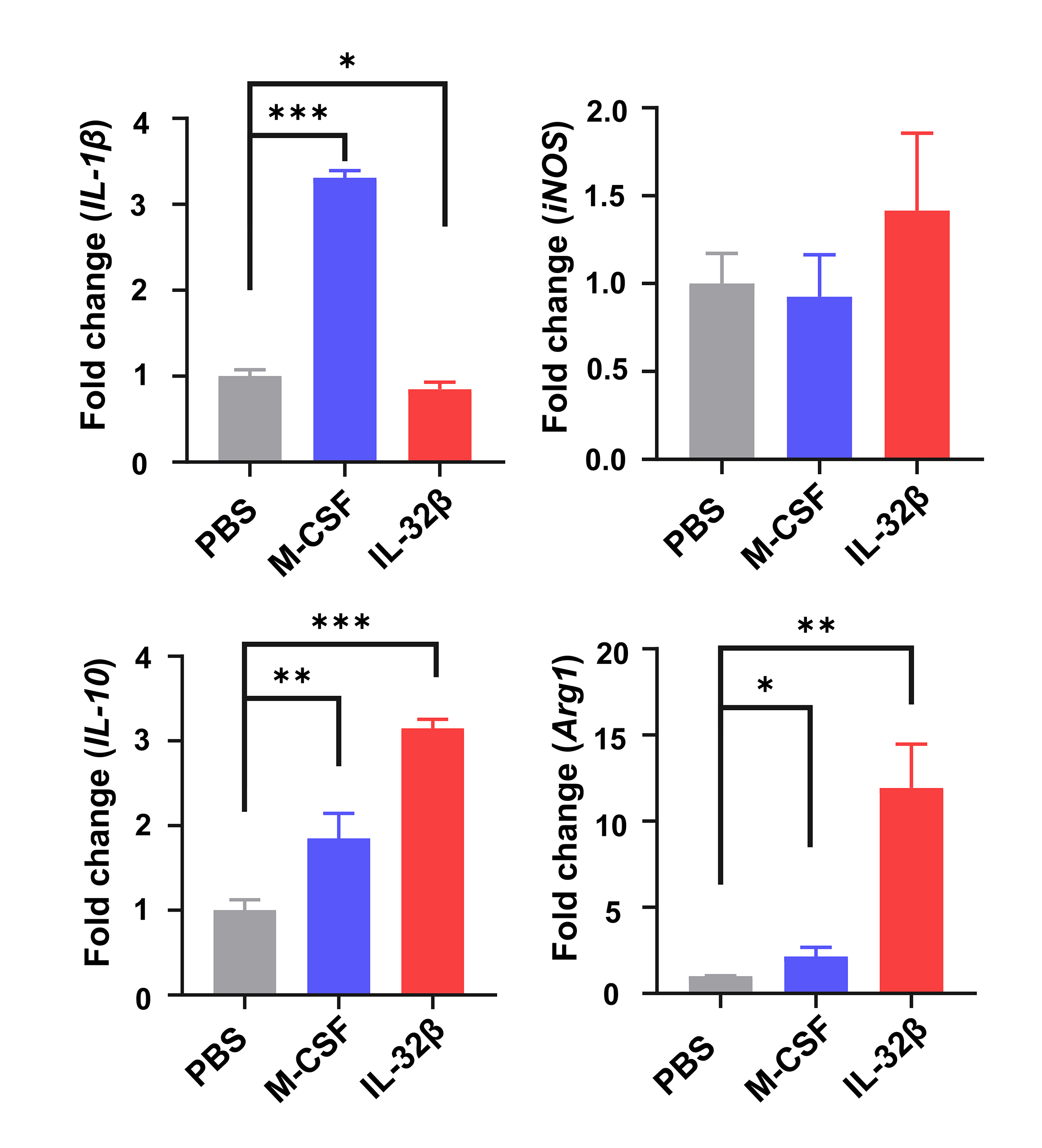


**Figure S12** MDMs were treated with recombination IL-32β and M-CSF at 50 ng/mL for 48 hours. qRT-PCR was performed to detect the expression of M2 macrophage-related genes (*IL-10, Arg1*) and M1 macrophage-related genes (*IL-1β, iNOS*). All data are representative of three independent experiments and represented as means ± SEM. Student’s *t*-test, **P* < 0.05, ***P* < 0.01, ****P* < 0.001.


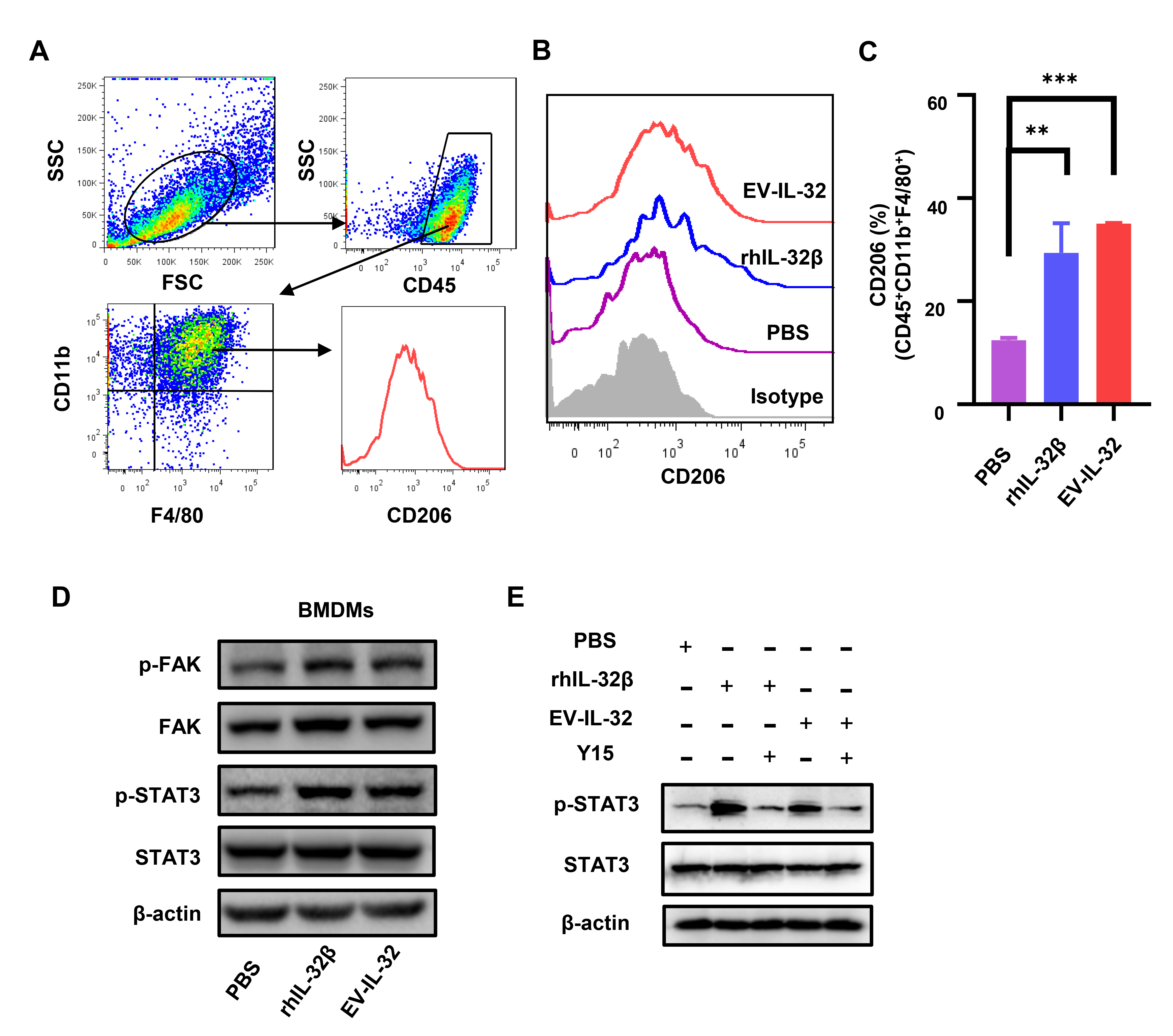


**Figure S13** Human IL-32 promotes mouse bone marrow-derived macrophages polarized to M2 macrophage. (A) Representative flow plots showing M2 macrophages (CD45^+^ F4/80^+^ CD11b^+^ CD206^+^). (B) BMDMs were induced by 20 ng/mL M-CSF (Peprotech, USA) for 7 days, and were treated with PBS, 50 ng/mL recombinant IL-32β or 50 μg/mL EV-IL-32 for 72 hours. The proportion of M2 macrophage was detected by flow cytometry, and the statistical (C) was shown. (D) Western blot assays for phosphorylated FAK and STAT3 expression using β-actin as control in mouse BMDMs treated with rhIL-32β (50 ng/mL) or EV-IL-32 (50 μg/mL) for 3 hours. (E) FAK inhibitor (Y15, 5 μM) decreased the phosphorylation of STAT3 in BMDMs treated rhIL-32β or EV-IL-32. The figures are representatives of three replicates. Scale bar, 100 μm. Student’s *t*-test, ***P* < 0.01, ****P* < 0.001.


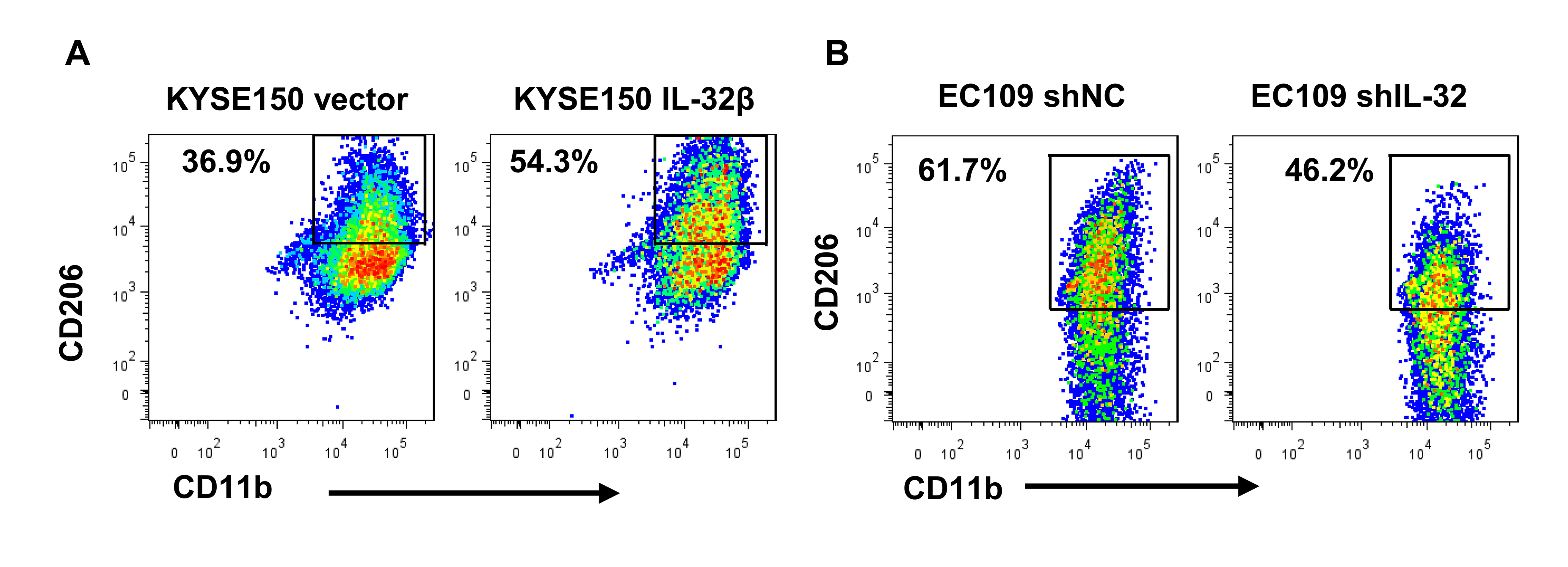


**Figure S14** Representative dot plots of intratumoral M2 macrophages were defined as CD45^+^F4/80^+^CD11b^+^ cells and the proportion was showed in mouse model of KYSE150 vector, KYSE150 IL-32β (A), EC109 shNC and EC109 shIL-32 (B).


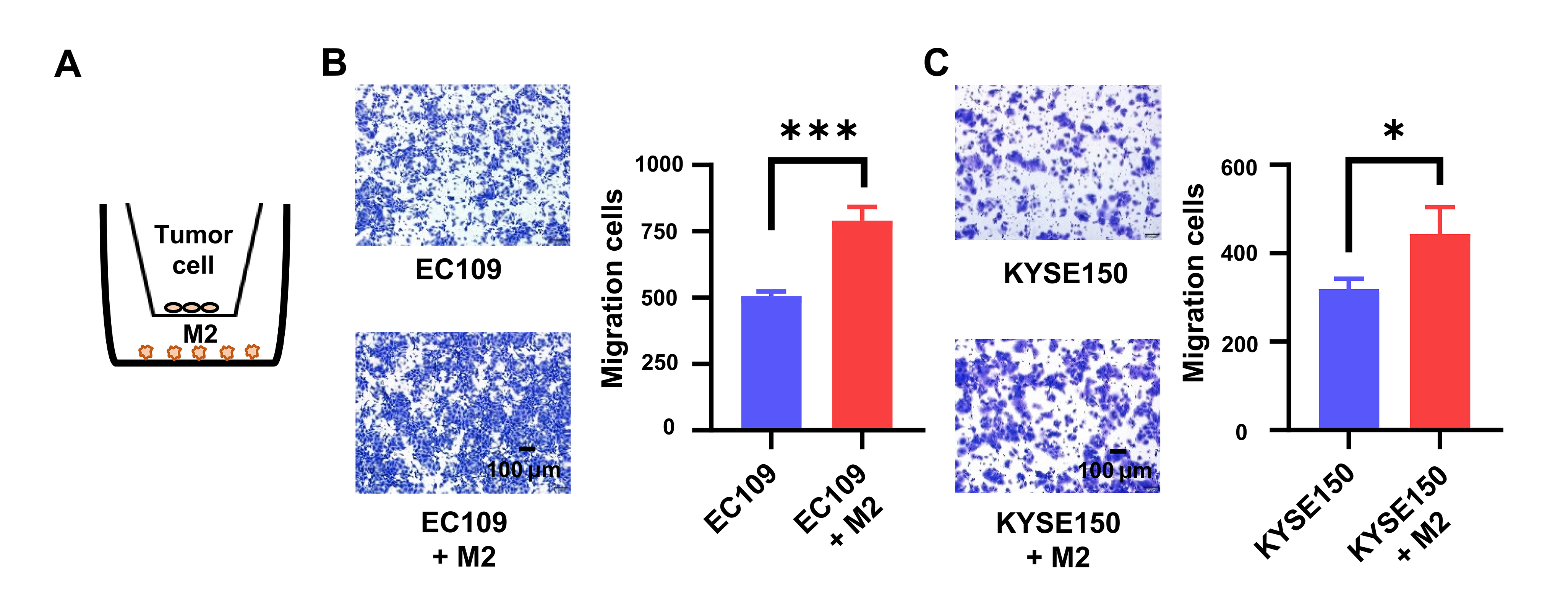


**Figure S15** EV-IL-32 educated M2 macrophage promotes metastasis of EC109 and KYSE150. (A) The coculture system proved that EV-IL-32 induced M2 macrophages promoted migration of ESCC cells *in vitro*. (B-C) EC109 and KYSE150 were seeded into the upper chamber with 1×10^5^/well and EV-IL-32 educated M2 macrophages into the lower chamber with the same density. Five fields were included per assay, and the experiment was repeated three times. Scale bar, 100 μm. Data are represented as means ± SEM. Student’s *t*-test, **P* < 0.05, ****P* < 0.001.


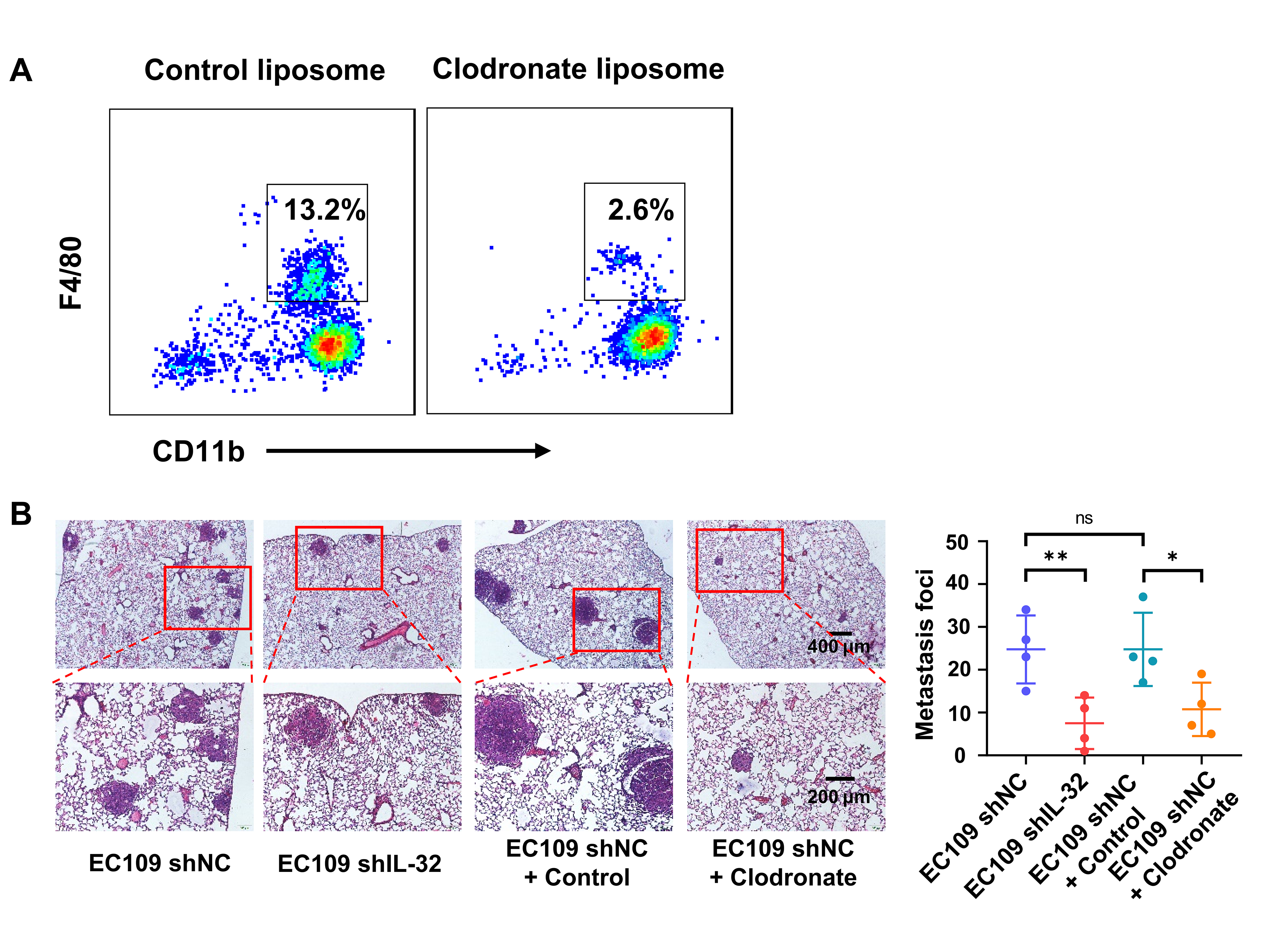


**Figure S16** Depletion of macrophages attenuating EC109 cells lung metastasis in vivo. (A) The percentage of F4/80^+^CD11b^+^ macrophages from blood were analyzed the day after administration of Control liposome or Clodronate liposome using flow cytometry. (B) Lung metastasis was confirmed by H&E (n = 4). Scale bars, 400 or 200 μm. Data are represented as means ± SEM. Student’s *t*-test, **P* < 0.05, ***P* < 0.01.


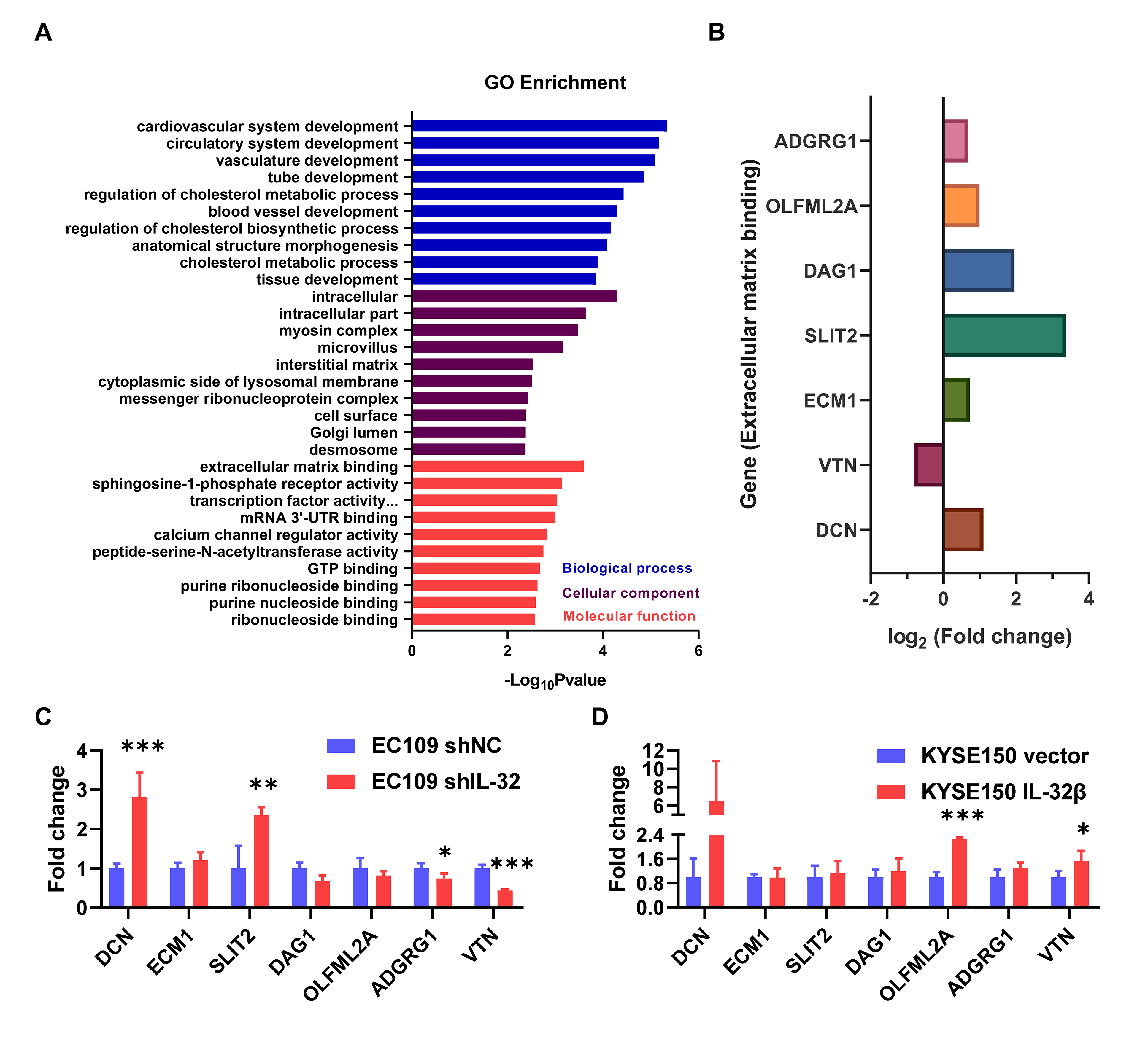


**Figure S17** The global transcriptional profile of EC109 shNC and EC109 shIL-32 was assessed by RNA-Seq. (A) The GO enrichment revealed that IL-32 knockdown in EC109 mainly affected vascular system development and extracellular matrix binding. (B) The data from RNA-seq was used to analyze the up-regulation and down-regulation genes in extracellular matrix binding. (C-D) qRT-RCR was performed to determine the fold change of genes about extracellular matrix binding in EC109 shNC, EC109 shIL-32, KYSE150 vector and KYSE150 IL-32β. Data are represented as means ± SEM. Student’s *t*-test, **P* < 0.05, ***P* < 0.01, ****P* < 0.001.

**Table S1** Primers for qRT-PCR to distinguish each isoform of IL-32.

| Gene | Forward | Reverse |
| --- | --- | --- |
| *IL-32*  *（all isoforms）* | ATGTGCTTCCCGAAGGTCCTCT | TCATTTTGAGGATTGGGGTTCA |
| *IL-32 γ* | CTACTTCTGCTCAGGGGTTGG | TGTGAAGAGAGGCAGAGTTGG |
| *IL-32 βγ* | ATGCAAAATGCAGAATCAGGACG | GGTGCTGCTCCTCATAATAAGCC |
| *IL-32 βγθ* | CTGTCCCGGATGTTGAGGAT | CACCACCTTCTCCTTCACCC |

**Table S2** Primers for qRT-PCR to amplify genes.

| Gene | Forward | Reverse |
| --- | --- | --- |
| *IL-32* | GGAGACAGTGGCGGCTTAT | GGCACCGTAATCCATCTCTT |
| *IL-10* | CGAGATGCCTTCAGCAGAGT | CGCCTTGATGTCTGGGTCTT |
| *IL-1β* | CTCGCCAGTGAAATGATGGCT | TAGTGGTGGTCGGAGATTCG |
| *iNOS* | CGCATGACCTTGGTGTTTGG | CATAGACCTTGGGCTTGCCA |
| *Arg1* | GTGGACAGACTAGGAATTGGCA | GGTCCAGTCCGTCAACATCA |
| *GAPDH* | GGAGTCCCTGCCACACTCA | GCCCCTCCCCTCTTCAAG |

**Table S3** Primary antibodies for FACS.

| Antibody | Company, catalog number |
| --- | --- |
| Anti-human CD14, APC | Thermo Fisher Scientific, 17-0149-42 |
| Anti-human CD11b, eFluor 450 | Thermo Fisher Scientific, 48-0112-80 |
| Anti-human CD80, PerCP-eFluor 710 | Thermo Fisher Scientific, 46-0809-42 |
| Anti-human CD206, FITC | BioLegend, 321104 |
| Anti-mouse CD45, FITC | Thermo Fisher Scientific, 11-0451-85 |
| Anti-mouse F4/80, eFluor 450 | Thermo Fisher Scientific, 48-4801-82 |
| Anti-mouse CD11b, PerCP-Cyanine5.5 | Thermo Fisher Scientific, 45-0112-82 |
| Anti-mouse CD11c, APC | Thermo Fisher Scientific, 17-0114-82 |
| Anti-mouse CD206 PE | Thermo Fisher Scientific, 12-2061-82 |

**Table S4** Primary antibodies for western blot.

| Antibody | Dilution | Company, catalog number |
| --- | --- | --- |
| IL-32 | 1: 2000 | BioLegend, 513501 |
| STAT1 | 1: 2000 | Sangon, D155186 |
| p-STAT1 | 1: 2000 | CST, 7649 |
| STAT3 | 1: 2000 | CST, 4904 |
| p-STAT3 | 1: 2000 | CST, 9145 |
| STAT6 | 1: 2000 | Sangon, D220089 |
| p-STAT6 | 1: 2000 | CST, 9361 |
| TSG101 | 1: 2000 | abcam, ab125011 |
| CD63 | 1: 2000 | abcam, ab134045 |
| FAK | 1: 2000 | Sangon, D121859 |
| p-FAK | 1: 2000 | Sangon, D155172 |
| GAPDH | 1: 2000 | Servicebio, GB11002 |
| β-actin | 1: 2000 | Servicebio, GB11001 |
